# Gap junction-mediated cell-to-cell communication in oral development and oral diseases: a concise review of research progress

**DOI:** 10.1038/s41368-020-0086-6

**Published:** 2020-06-12

**Authors:** Wenjing Liu, Yujia Cui, Jieya Wei, Jianxun Sun, Liwei Zheng, Jing Xie

**Affiliations:** State Key Laboratory of Oral Diseases & National Clinical Center for Oral Diseases & West China Hospital of Stomatology, Sichuan University, Chengdu, China

**Keywords:** Adherens junctions, Focal adhesion

## Abstract

Homoeostasis depends on the close connection and intimate molecular exchange between extracellular, intracellular and intercellular networks. Intercellular communication is largely mediated by gap junctions (GJs), a type of specialized membrane contact composed of variable number of channels that enable direct communication between cells by allowing small molecules to pass directly into the cytoplasm of neighbouring cells. Although considerable evidence indicates that gap junctions contribute to the functions of many organs, such as the bone, intestine, kidney, heart, brain and nerve, less is known about their role in oral development and disease. In this review, the current progress in understanding the background of connexins and the functions of gap junctions in oral development and diseases is discussed. The homoeostasis of tooth and periodontal tissues, normal tooth and maxillofacial development, saliva secretion and the integrity of the oral mucosa depend on the proper function of gap junctions. Knowledge of this pattern of cell–cell communication is required for a better understanding of oral diseases. With the ever-increasing understanding of connexins in oral diseases, therapeutic strategies could be developed to target these membrane channels in various oral diseases and maxillofacial dysplasia.

## Introduction

Cell–cell communication is vital for cell differentiation, morphogenesis, cell growth and homoeostasis in multicellular organisms.^1^ It was described as “the music that the nucleus hears”, and once it is dissonant, abnormal communication between cells may disrupt biological processes.^2^ The indispensable components of cell–cell communication include tight junctions, anchoring junctions (adherens, desmosomes, focal adhesions and hemidesmosomes) and communication junctions (gap junctions, pannexins, ion channels, and chemical synapses).^1^ Gap junctions are clusters of intercellular channels facilitating a direct connection between the cytoplasm of two neighbouring cells to mediate intercellular communication.^3^ These channels are formed by channel-forming proteins that are densely packed into spatial microdomains of the plasma membrane. Three families of channel-forming proteins have been identified, i.e., innexins, connexins and pannexins, among which innexins are located in the protostome and the other two families are present in deuterostomes. The pannexin family is considered a special type of channel-forming proteins that functions as a hemichannel.^4^ The connexin family exists in the form of hemichannels and assembles into gap junctions in vertebrates. In 1952, Weidman described gap junctions in the myocardium, and thereafter Furshpan and Potter detected these in neurons.^5,6^ Currently, 21 connexins have been identified in humans and 20 have been detected in the mouse genome. Gap junctions play pivotal roles in a wide range of physiological processes, including electrical activation of the heart,^7^ neuronal signalling,^8^ hormone secretion,^9^ auditory function,^10^ wound healing,^11^ immune functions,^12^ inflammatory disorders^13^ and bone remodelling.^14^ Moreover, gap junctions promote the brain metastasis of carcinoma into astrocytes through cGAMP transfer.^15^ The oral cavity and its appendices are exposed to an intricate environment and considerable mechanical stress.^16^ Tight polar connections between epithelial cells, which protect the oral mucosa from microbial infections and mechanical stress, are generated from various cell–cell and cell–extracellular matrix junctions.^17^ However, compared with the well-discussed roles in physiological processes, the functions of gap junctions in oral tissues under healthy and pathological conditions remain to be further explored. Thus, in this review, we have focused on the roles of gap junctions in oral development and oral diseases.

## Gap junctions formed by connexins

All junctional channels have an analogous integral structure. However, unlike other membrane channels, different gene families encode the membrane proteins that form junctional channels in different animal phyla.^18^ In vertebrates, the corresponding genes are denominated with a symbol beginning with “*GJ*” (for gap junction), which represents a virtual narrow separation of 2–4 nm between two neighbouring cells observed using a transmission electron microscope (TEM).^19^ Meanwhile, the proteins are generally referred to with an abbreviation beginning with “Cx” (for connexin) combined with a number corresponding to the approximate molecular mass of the predicted polypeptide in kilodaltons (kDa).^20^

### Structure and function of the connexin hemichannel

#### Structure of connexins

**Fig. 1:**
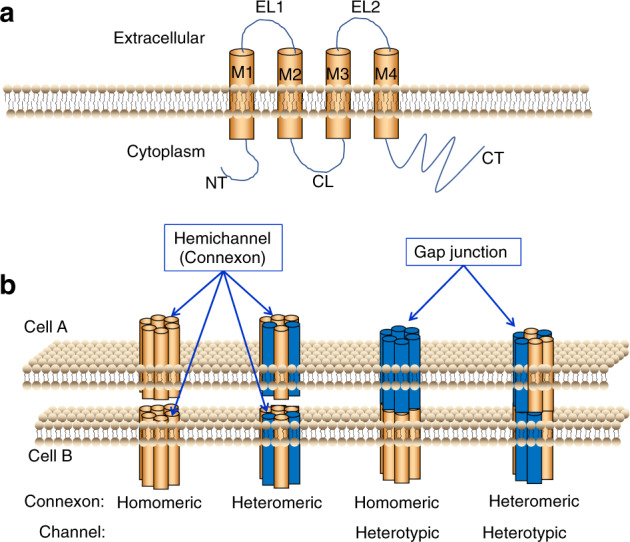
Model of a connexin and gap junction channel. **a** The connexin monomer. NT, N-terminus; CL, cytoplasmic loop; CT, C-terminus; EL1 and EL2, extracellular loops 1 and 2; M1–M4, transmembrane domains 1–4. **b** Possible arrangements of connexins in a gap junction channel. The figure shows different components of gap junction channels. Homomeric connexons are formed by a single connexin type. Heteromeric connexons are composed of more than one connexin type. When connexons of the same composition form a gap junction channel, it is classified as a homotypic channel. If the connexons differ in components, the channel is classified as heterotypic

**Fig. 2:**
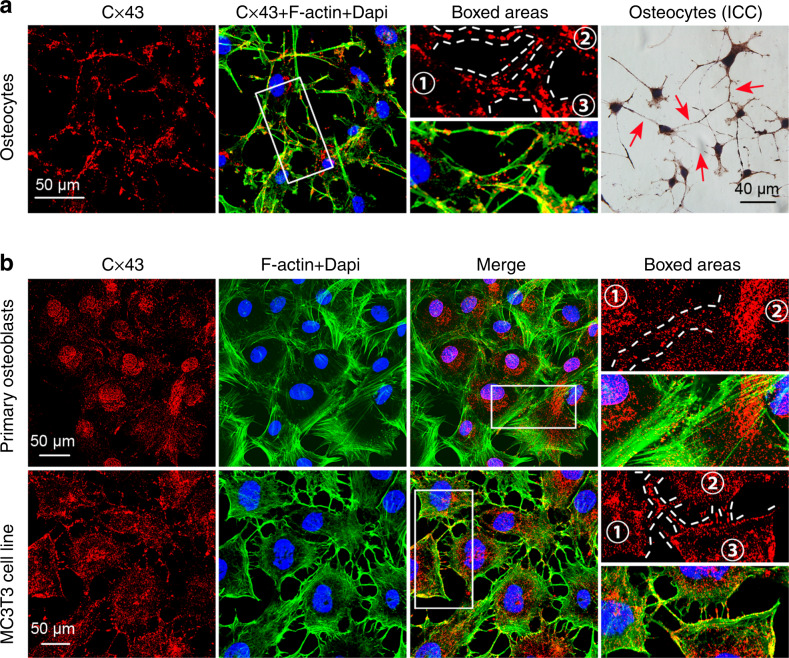
Distribution of Cx43 in osteocytes and osteoblasts. **a** Representative images of IF staining for Cx43 in osteocytes. Immunofluorescence staining was performed with antibodies against connexin 43 (red); nuclei were stained with DAPI (blue). Green represents the FITC-labelled cytoskeleton. Details are shown in the boxed area (white). Representative ICC staining of osteocytes. The red arrow indicates the localization of Cx43. **b** Representative images of IF staining for Cx43 in osteoblasts. Representative image of IF staining for Cx43 in primary osteoblasts. Representative image of IF staining for Cx43 in the MC3T3 cell line. Immunofluorescence staining was performed with antibodies against connexin 43 (red); nuclei were stained with DAPI (blue). Green represents the FITC-labelled cytoskeleton. Details are shown in the boxed area (white)

Each connexin protein is composed of four transmembrane α-helices (TM1–TM4) connected by two extracellular loops and one intracellular loop.^21^ Their long C-termini (CT) and short N-termini (NT) are located on the cytoplasmic side of the membrane (Fig. 1a). Three cysteine residues reside in each of the two extracellular loops and a proline residue is strictly conserved in connexin proteins, which are critical for intramolecular stabilization.^18,22^ Currently, connexins are categorized into five subfamilies, i.e., α, β, γ, δ and ε or GJA, GJB, GJC, GJD and GJE, according to the differences and similarities in the amino acid sequences.^20^ The distribution of connexins (Cxs) varies according to the cell types, the developmental period and the species, partially due to their trafficking towards the plasma membrane after synthesis in the endoplasmic reticulum. Connexin 43 (Cx43) is also expressed on mitochondrial membranes and is called mitochondrial Cx43 (mito Cx43).^23^ As shown in our previous study, Cx43 localizes in the cytoplasm and dendritic processes of osteocytes and even clusters as plaques at the intersection of the dendritic processes of two cells (Fig. 2a).^24^ We also observed a scattered, punctate distribution of Cx43 at the sites of cell–cell contacts in osteoblast (Fig. 2b).

#### Connexin phosphorylation

The connexin “lifecycle” is a complex process involving the transcription of a specific connexin gene, trafficking, assembly, synthesis and turnover. Several phosphorylation events at multiple sites on connexins have been linked to GJ internalization and turnover. Functional data on phosphorylation has been reported for Cx32, Cx43, Cx45 and Cx56. The C-terminal region of the connexin proteins appears to be the main region that is phosphorylated, except for Cx56, which also contains phosphorylation sites within the cytoplasmic loop region, in addition to its C-terminal domain.^25,26^ No reports have identified phosphorylation sites at the N-terminus of connexins. Cx26 is the only connexin that exists in a non-phosphorylated state.^27^ Notably, connexins have rapid turnover rates as membrane proteins. For instance, the half-life of Cx32 in rodent hepatocytes is <5 h in vivo.^28^ The phosphorylation of Cx43 at different sites also controls gap junction degradation.^29^ According to Fernandes et al., Cx43 phosphorylation attenuates the assembly of gap junctions and potentially leads to Cx43 degradation.^30^ Moreover, connexin phosphorylation is also associated with the gating of hemichannels or intact gap junction channels. When Cx43 is phosphorylated at the MAPK sites in the presence of a normal extracellular [Ca^2+^] concentration, connexin hemichannels are closed. However, dephosphorylation of those sites by phosphatases in response to a biological stressor, such as hyperosmolarity, induces opening of the hemichannels, resulting in the influx of extracellular ions that impair the function of cells.^31^ Phosphorylation of Cxs is very important in regulating gap junctional intercellular communication (GJIC), several other sites have been identified and additional phosphorylation sites will likely be identified in the future.

### Gap junctions

#### Structure of gap junctions

Six connexin subunits are arranged into a hexamer, forming an annular torus structure around an aqueous pore, which is called a hemichannel or a connexon. Connexons are classified into homomeric (composed of a single connexin protein) and heteromeric (composed of two or more different connexins) channels based on the composition of the channel. Those connexons are then transported to the cell membrane surface, where they dock with a partner connexon from an adjacent cell, forming an intercellular channel that spans the two cells and is called a gap junction channel.^32^ In addition to homomeric, homotypic channels, a diverse arrangement of gap junctional channels exists between apposed cells. The gap junction channel is classified as homotypic when it is formed from connexons with the same composition. Conversely, if the components of the connexons differ, the gap junctional channel is defined as heterotypic (Fig. 1b).^33^

#### Function of gap junctions

Gap junctions display a relatively low substrate specificity and are permeable to a wide variety of molecules with mass <1 kDa, such as small metabolites, ions and intracellular signalling molecules (i.e., various ions, ATP, ADP, cAMP, amino acids, small peptides, glucose, inositol triphosphate, cyclic nucleotides and oligonucleotides).^34^ Gap junctional intercellular communication is actively involved in virtually all aspects of the cellular life cycle, ranging from cell growth, differentiation and function to cell death. The transfer of current and electrical coupling between cells are key factors regulating the function of excitable tissues, e.g., the heart, in which rapid current transmission is mediated by gap junctional channels between adjacent cells.^7^ In addition to the heart, gap junctions provide a direct pathway of low resistance for the spread of presynaptic electrical currents to the postsynaptic site in electrical synapses of neurons.^35^ Moreover, gap junctions facilitate the diffusion of signals from various molecules. Based on accumulating evidence, some Cx channels are permeable to certain soluble second messengers, amino acids, nucleotides and glucose and its metabolites.^18^ Furthermore, cell-to-cell propagation of calcium waves, which may be initiated by a focal mechanical, electrical or hormonal stimulus, serve to coordinate a global cellular response by diffusing IP_3_ through the gap junctions between cells.^36^

#### Regulatory effects of growth factors on gap junctions

Given the diversity of gap junction structures, the mechanisms controlling gap junction activity are complex. Various factors are involved in the regulation of GJIC, e.g., growth factors and changes in extracellular matrix.^37,38^ Several growth factors have been confirmed to be involved in the regulation of GJIC, and different growth factors induce distinct and even opposite effects on gap junctions.

The effect of epidermal growth factor (EGF) on GJIC of different cell types has been reported. In most cases, EGF reduced the intercellular communication via GJs. For instance, the application of 10 ng/ml epidermal growth factor (EGF) for 24 h reduced GJIC in human keratinocytes, and this inhibitory effect of EGF was induced by the MAPK-mediated phosphorylation of Cx43 at Ser255.^39^ In addition, EGF decreased the expression of the Cx43 protein in cultured rat cortical astrocytes.^40^ However, in some cell types, such as the K7 human kidney epithelial cell line and granulosa cells, EGF increased the amount of the Cx43 transcript and protein, as well as the function of GJs.^41,42^ In addition to affecting the functional state of Cx43-mediated GJs, EGF has been reported to modulate other Cx proteins, such as Cx32, which is upregulated by EGF in hepatocytes.^43^

Controversies exist regarding the effect of platelet-derived growth factor (PDGF) on GJs. The addition of PDGF to mesangial cell cultures causes a rapid and transient inhibition of GJIC, with maximal inhibition occurring 15 min after PDGF exposure and returning to control levels after 90 min.^44^ After the transfection of T51B cells, a rat liver epithelial cell line lacking endogenous PDGF receptors, with a retrovirus encoding wild type human PDGF receptor, GJIC was completely and transiently interrupted upon treatment with PDGF.^45^ This induced change was associated with increased Cx43 phosphorylation and MAPK activation. PDGF does not affect GJIC in cells transfected with Cx43 truncated at amino acid 256 and carrying a myc tag appended to its C-terminus, suggesting that a Cx43 target site is related to the reduction in GJIC.^46^ However, PDGF was recently shown to increase Cx43 expression under hypoxic conditions.^47^

Similar to PDGF, fibroblast growth factor-2 (FGF-2) was shown to exert a biphasic effect on GJIC in cardiac fibroblasts, reducing it within 30 min (short-term effect), but increasing it after longer periods (>6 h).^48^ FGF-2 also reduced the levels of the Cx43 transcript and protein in astroglial cells.^49^ This FGF-2-induced reduction was likely due to the PKC-mediated phosphorylation of Cx43 at serine 368.^50^ In cardiomyocytes, FGF signalling was shown to be essential for Cx43 phosphorylation and cardiac gap junction maintenance.^51^ FGF-5 and FGF-9 induce a decrease in the number of gap junctions in specific brain regions.^49^ As shown in our previous study, FGF-7 induces the expression of Cx43 and enhances the function of GJs in osteocytes.^52^ Thus, members of the fibroblast growth factor family affect GJIC in a complex manner.

According to our recent studies, transforming growth factor-beta 1 (TGF-β1) also enhances GJIC by upregulating the level of the Cx43 protein in osteocytes and chondrocytes.^53–55^ The mechanism by which TGF-β1 increases Cx43 expression is through the activation of the ERK and Smad signalling pathways.^53^ However, the role of TGF-β1 in modulating the expression of the Cx43 transcript and protein also depends on the cell type. TGF-β1 upregulates Cx43 expression in human granulosa cells and trophoblast cells.^56,57^ In contrast, TGF-β1 downregulates Cx43 expression in cultured smooth muscle cells from the human detrusor and in rat hepatic stellate cells.^58,59^ TGF-β2 was recently shown to induce the expression of the Cx43 protein in MSCs.^60^ TGF-β3 was also shown to increase gap junctional communication among folliculostellate cells.^61^ Additional studies are required to elucidate the complicated effects of growth factors on GJs in the future.

#### The protein interaction partners of connexins

Based on accumulating evidence obtained in recent years, gap junction proteins do not act as isolated entities in the plasma membrane, but rather interact with a series of partner proteins that link them to the cytoskeleton and to signalling pathways. Multiple proteins have been reported to interact or only colocalize with gap junction proteins.^62^ Gap junctions have only recently been reported to interact with the actin cytoskeleton. Squecco et al.^63^ observed the colocalization of Cx43 and F-actin in C2C12 cells. Cx43 was also suggested to interact with α-tubulin and β-tubulin.^64^ Zonula occludens (ZO) proteins, one ubiquitous type of scaffolding protein, may play a general role in the formation and turnover of gap junctions to regulate intercellular communication, since all Cxs identified to date have been reported to interact with ZO proteins. For example, ZO-1 directly tethers connexins to the actin skeleton,^65^ and ZO-2 also binds to the C-terminus of Cx43.^66^ However, functional GJs are formed even when the interaction between connexins and ZO-1 is blocked, suggesting that other mechanisms are also involved in plaque formation.^67^

Gap junctions are formed through cell–cell contact and homophilic cadherin–cadherin interactions.^68^ Occludin, a protein involved in the formation of tight junctions, interacts with Cx32 in immortalized mouse hepatocytes.^69^ As shown in the study by Nusrat et al., Cx26 interacts with the coiled-coil domain of occludin in epithelial cells.^70^ Claudins are another component of the tight junction complexes that have a similar topology to Cxs. Claudin-1 colocalizes with Cx32 in rat hepatocytes lines,^71^ and claudin-5 coprecipitates with Cx43 in porcine blood-brain barrier endothelial cells.^72^ In addition to the proteins forming tight junctions, N-cadherin, the core component of adherens junctions, also plays a central role in mediating cell–cell interactions. In NIH3T3 cells, Cx43 interacts with an N-cadherin-containing multiprotein complex. Moreover, this interaction has been shown to be essential for gap junction formation.^73^ When the N-cadherin gene is deleted in N-cadherin knockout mice, cardiomyocytes are deficient gap junctions.^74^ However, an ongoing controversy exists over the role of N-cadherin in the formation of gap junction. According to Govindarajan et al.^75^, the expression of N-cadherin attenuates GJ assembly in rat liver epithelial cells by inducing the endocytosis of Cx43.

Some membrane channels and enzymes are also connexin partners. Sodium channel complexes interact with Cx43 in ventricular myocytes.^76^ Aquaporin, a member of the major intrinsic protein (MIP) superfamily, interacts with two binding sites within the intracellular loop region of Cx50.^77^ The long cytosolic C-terminus (CT) of Cx43 is required for the proper function of Cx43 gap junctions. The C-terminus of several connexins (e.g., Cx43) contains consensus phosphorylation sites for several proteins, including Src protein tyrosine kinases,^78^ Akt^79^ and mitogen-activated protein kinase (MAPK).^80^ Protein kinase C (PKC) displays partial colocalization with Cx43 and directly phosphorylates Cx43 at Ser368,^81^ thus modulating various phases of the Cx43 life cycle, including gap junction assembly, gap junction channel permeability, and Cx43 endocytosis and degradation. Conceivably, the interactions between Cxs and other proteins have important functions under physiological conditions and in associated diseases, such as oral disease.

## Connexins in oral development and oral diseases

### Connexins in oral and maxillofacial development

#### Connexins in tooth development

Tooth development depends on the sequential and reciprocal interactions between the epithelium and mesenchyme. In the developing tooth germ of neonatal rats, Cx43 is distributed both in epithelial and mesenchymal dental cells;^82^ Cx43 has been detected between ameloblasts and between odontoblasts.^83,84^ Cx43 expression gradually increases with the progression of odontoblast maturation from pre-odontoblast to old odontoblasts.^85^ During ameloblast development, Cx43 expression shows a transient decrease in the late presecretory ameloblasts before enamel formation and then increases during the secretory stage.^86^ At the maturation stage of enamel, GJs contribute to enamel formation by transporting ions from the papillary layer to ameloblasts.^87^ As shown in the study by Toth et al.^88^, dominant negative G60S mutants of Cx43 result in ameloblast dysregulation and enamel hypoplasia. Once tooth germ development is completed, consistent expression of Cx43 at high levels in human dental follicle cells (HDFCs) is essential for tooth eruption.^89^ In addition to the involvement of Cx43 in enamel development, Cx32 has also been detected in the developing enamel organ.^90^ Based on these findings, Cxs are involved in tooth development, and a certain connexin may have distinct roles in odontogenesis and tooth homoeostasis.

#### Connexins in maxillofacial development

Cx43 plays a critical role in the development of maxillofacial structures. Patients who suffer from oculodentodigital dysplasia (ODDD), which is caused by mutations in *Cx43/GJα1*, present oral dysfunction, including oral clefting, numerous cavities and tooth loss.^91^ Cx43 and Cx32 are related to differentiation and growth during the early phase of submandibular gland development. During this period, Cx43 is considered to contribute to the branching morphogenesis^92^ and the contractile function of myoepithelial cells, while Cx32 expression may correspond to an increase and decrease in the number of proacinar and mature acinar cells, respectively.^93^ Cx43 also plays an important role in the initiation of papillary pattern formation and morphogenesis in the tongue.^94^ For instance, Cx43 regulates the localization of keratins to pre-pattern of the bud region during the formation of the circumvallate papilla.^95^ Compared with the available data on the functions of Cxs in tooth development, much less is known about the roles of Cxs and GJs in maxillofacial development, such as palate and mandible development.

### Connexins in oral diseases

#### Connexins in periodontal tissue

The periodontal ligament (PDL) is a soft connective tissue that resides between the alveolar bone and tooth to sustain teeth and preserve tissue homoeostasis. Periodontal tissue homoeostasis depends on a complicated cellular network that transmits signals between periodontal ligament fibroblasts (PDLFs).^96^ Cx proteins (Cx32, Cx40, Cx43 and Cx45) are expressed in PDLFs,^97,98^ while Cx26 and Cx37 have not been detected. Those proteins have two main functions. One is the correlation between Cx32 and the secretory function of PDLFs, i.e., the ability to produce type I and type III collagen and fibronectin. On the other hand, Cx40 and Cx45 are considered to relate to the contractile function of PDLFs, which may relate to tooth eruption.^99^ Moreover, Cx43 is involved in the transmission of signals from mechanical stimuli in the PDL. During orthodontic tooth movement, Cx43 expression is increased in rat PDLFs.^100^ Mechanical tension also increases the expression of Cx43 and promotes GJIC in a time-dependent manner, while 24 h cyclic stretches downregulate the expression of Cx43 in the membrane of hPDLFs.^98,101^ Cxs have also been studied in human gingival epithelium, where keratinocytes normally express Cx26 and Cx43 at high levels,^102–104^ and Cx43 even forms plaques in fibroblasts. In the rat gingival epithelia, Cx43 was detected in the basal layer and middle of the prickle cell layer. Interestingly, in the human gingival epithelia, Cx43 expression showed a progressive decrease from the spinous layers of the oral gingival epithelium to the sulcular epithelium and parts of the junctional epithelium.^102,103^ Cx26 was detected in the granular cell layer and lower part of the squamous cell layer.^105^ Nevertheless, Cx43 was downregulated at the early stage of gingival wound healing, which may contribute to the fast wound healing of the gingival tissue.^106^ However, to the best of our knowledge, no information is available on the role of gap junctions in periodontal disease.

#### Connexins in tooth development and diseases

Dental pulp contains fibroblasts, odontoblasts and undifferentiated mesenchymal cells, and dental pulp fibroblasts (DPFs) are major components among these cells.^107^ Cx32 and Cx43 expression have been observed in human DPFs. In cultured human pulp cells, Cx43 expression is upregulated during mineralization processes, indicating that Cx43 might play a role in mineralization.^82^ This finding was further supported by a study showing that the inhibition of Cx43 attenuates the odontoblastic differentiation and mineralization of rat dental pulp cells.^108^ In addition, Shiting et al.^109^ found that the overexpression of Cx43 potentiated the odontoblastic differentiation of dental pulp stem cells (DPSCs), suggesting that Cx43 is involved in the differentiation of DPSCs into odontoblasts. Cx43 is a marker of the viability of dental pulp tissue, as Cx43 expression is reduced in aged human dental pulp. A reduction in Cx43 expression may be one characteristic of aged pulp.^110^ This hypothesis was further verified by the rapid degradation of Cx43 in pulp cells exposed to physical stimuli such as heat.^111^ In intact adult teeth, Cx43 forms gap junctions between odontoblasts.^112^ Microscopic observations indicate the localization of gap junctions between the bodies of odontoblasts and between the bodies and processes of odontoblasts.^113^ Similarly, Cx26 is expressed at low levels in the odontoblast layer, while Cx32 is not detected in odontoblasts.^90^ In carious teeth, Cx43 expression is upregulated in mature odontoblasts in the vicinity of carious lesions, combined with the prominent expression of Cx43 in the interodontoblastic cells.^114^ However, the expression of Cx43 between odontoblasts is reduced in reactionary dentin that forms during dentin caries development.^115^ Notably, fluoride, which is recognized to protect against tooth decay, increases Cx43 and Cx32 expression, but decreases Cx45 expression in rat incisor pulp.^116^ Nevertheless, the scientific evidence supporting the correlation between Cxs and caries is insufficient, and the mechanism by which fluoride regulates connexin expression in odontoblasts remains to be elucidated.

#### Connexins in oral cancer

Connexins may be associated with cell growth, since the absence of GJIC can result in an accumulation of growth factors in cells^117^ and a suppression of contact inhibition, which together lead to cell proliferation.^118^ In addition, a decrease in Cx43 and Cx32 expression may result in the uncontrolled proliferation and abnormal differentiation of various benign and malignant tumour cells.^119–121^ Oral squamous cell carcinoma (OSCC) is the most prevalent and most commonly studied oral cancer.^122^ However, the potential role of the oral microbiome in OSCC has not been clearly elucidated. In the dysplasia-free oral mucosa, Cx43 is mainly expressed on the membrane in the stratum spinosum and stratum granulosum, but is not expressed in the stratum corneum. However, a significant increase in cytoplasmic Cx43 expression and a loss of GJIC have been observed during the early carcinogenesis of OSCC. Therefore, membrane Cx43 levels might be an independent biomarker for early changes associated with oral squamous cell carcinoma.^123^ Moreover, Cx43^+^ fibroblasts are enriched in the stroma of OSCC, which may be a hallmark for judging oral SCC invasion.^124^ Changes in Cx43 expression have been detected in malignant and benign tumours. In another study, the downregulation of Cx43 and Cx32 expression was observed in keratocystic odontogenic tumours, one of the most frequently occurring types of benign odontogenic tumours.^125^ In addition to studies of changes in Cx43 and Cx32 expression during carcinogenesis, Cx26 expression was also reported to be reduced in tongue carcinoma.^126,127^ Recently, many studies have focused on therapies that restore connexin-mediated GJIC in oral cancer. For example, all-*trans* retinoic acid was shown to be beneficial for OSCC cells to regain cell–cell communication by increasing Cx32 and Cx43 expression.^128^ According to Marie et al., gap junctional intercellular communication is enhanced by docetaxel in salivary gland carcinoma, concomitant with an increase in Cx43 expression and its membrane localization.^129^ Lycopene significantly increased Cx43 expression and GJIC between KB-1 cells, which originate from a human oral cavity tumour.^130^ Based on the information described above, Cx43 may be a tumour suppressor and a potentially novel therapeutic target for oral cancer.

#### Connexins in diseases caused by gene mutations

Functional studies have begun to identify some of the underlying mechanisms by which connexin channel mutations contribute to oral cavity diseases. Keratitis–ichthyosis–deafness (KID) syndrome is a rare ectodermal dysplasia caused by mutations in the *GJB2* gene, which is responsible for the production of the Cx26 protein, a protein present in the epithelial gap junctions that is postulated to be associated with the differentiation of ectodermally derived tissues. Phenotypic features associated with Cx26 mutations are significant visual and auditory impairments. Affected patients are also at increased risk of developing epithelial malignancies. One KID syndrome has been noted to confer a predisposition to SCC.^131^ Approximately 11% of patients with KID syndrome develop this condition.^132^ Oculodentodigital dysplasia (ODDD) is another congenital disorder caused by a mutation in the *GJα1* gene, which encodes Cx43.^133^ It is characterized by multiple phenotypic abnormalities involving the face, limbs, teeth and eyes, as well as neurological symptomatology. Concerning the teeth, microdontia is present in one-fifth of the patients. More frequently, patients suffer from the amelogenesis imperfecta (AI), hypoplastic type. Other dental symptoms reported include malocclusion, delayed tooth development, pulp stones, tooth loss and missing teeth.^134^

#### Connexins and wound healing in the oral cavity

The suppression of Cx43 expression or function promotes skin wound healing and alleviates scarring. Corresponding to this finding, Cx43 expression is substantially decreased in human gingival fibroblasts at the early stage of wound closure, and Cx43 regulates the expression of wound-healing genes in gingiva. Thus, downregulation of Cx43 appears to be conductive to fast and scarless wound healing in gingival tissues.^106,135,136^ Consistent with this finding, Masato et al. also observed a wound-induced decrease and subsequent increase in Cx43 expression in the hamster tongue epithelium.^126^ Interestingly, in contrast to skin after injury, the expression of Cx43 in buccal mucosa wounds decreases and remains at a low level for 14 days. Increased Cx43 expression affects MMP-1 synthesis, which facilitates scar formation.^137^ Together, these studies explain why the oral mucosa is less prone to form a scar after wound healing compared with skin.

#### Connexins in the salivary gland

Salivary glands play an important role in oral biology by secreting saliva to provide water for lubrication, as well as electrolytes, mucus, enzymes and antibacterial compounds. Abnormal function of the salivary gland can lead to an extensive deterioration of oral health. Gap junctions have recently been suggested to be involved in maintaining salivary gland function.^138^ Cx26 and Cx32 colocalize within the same gap junctional plaque between acinar cells in rat parotid glands, but no expression of these two proteins is observed in the ducts.^139,140^ In rat submandibular and sublingual glands, Cx32 is distributed at the membranes between acinar cells and Cx43 is localized at the gap junctions between the thin processes of myoepithelial cells, suggesting that Cx32-meditated GJs are related to regulation of the secretory function of acinar cells and Cx43-mediated GJs are involved in the contractile function of myoepithelial cells.^141,142^ However, these studies are still limited to exploring phenotypes, and the role of gap junctions in specific salivary gland diseases is unknown. Therefore, an analysis of connexins and gap junctions will hopefully contribute to the study of salivary diseases.

## Conclusions and perspectives

As data documenting the functions of connexins in the oral health and oral disease are still limited, information is mainly confined to the distribution of Cxs in diverse oral tissues during different developmental phases. Far fewer reports have described the role of functional GJs in oral diseases such as periodontitis and chronic apical periodontitis. Previous studies have provided support for additional roles of Cxs in oral development and the pathogenesis and prognosis of oral diseases. Studies of various Cxs in oral tissues are categorized in Table 1. Collectively, Cxs and GJs play important roles in maintaining the normal development and function of oral tissues. Specific Cxs may potentially represent molecular targets for the treatment of certain oral diseases. Therefore, Cxs and gap junctions appear to be a very interesting field for additional research.

**Table 1:** The reports on connexins that we can currently collect

Protein	Gene name		Oral cavity-related distribution, development and disorders	References
	Human	Mouse		
Cx26	*GJB2*	*Gjb2*	Gingival	^102,105^
			Odontoblast	^90^
			Tongue carcinoma	^126,127^
			KID	^131,132^
			Salivary gland	^139,140^
Cx32	*GJB1*	*Gjb1*	PDLFs	^99^
			Pulp	^116^
			Keratocystic odontogenic tumours	^125^
			Salivary gland	^93,139,140,142^
Cx40	*GJA5*	*Gja5*	PDLFs	^99^
Cx43	*GJA1*	*Gja1*	PDLFs	^98,100,101^
			Gingiva	^102–104,106,135,136^
			Pulp cell; DPSCs; pulp	^82,108–111,116^
			Odontoblast	^84,112–115^
			ODDD	^91,133,134^
			Tongue	^94,95^
			Ameloblasts; enamel hypoplasia	^86–88^
			HDFCs	^89^
			OSCC; KCOTs	^123–125,128^
			Buccal mucosa	^137^
			Salivary gland	^92,142^
Cx45	*GJA7*	*Gja7*	PDLFs	^99^
			Pulp	^116^

KID, keratitis–ichthyosis-deafness; PDLFs, periodontal ligament fibroblasts; DPSCs, dental pulp stem cells; ODDD, oculodentodigital dysplasia; HDFC, human dental follicle cells; OSCC, oral squamous cell carcinoma; KCOTs, keratocystic odontogenic tumours

## Supplementary information

SUPPLEMENTAL MATERIAL
