# Supplementary material for: Gap junction-mediated cell-to-cell communication in oral development and oral diseases: a concise review of research progress
Source: Int J Oral Sci. 2020 Jun 12;12:17. doi: 10.1038/s41368-020-0086-6 (PMC7293327; doi:10.1038/s41368-020-0086-6)
Supplement: Supplementary file 1 — SUPPLEMENTAL MATERIAL [file 41368_2020_86_MOESM1_ESM.doc]

**Title page**

*Review article*

**Gap junction-mediated cell-to-cell communication in oral development and oral diseases: a concise review of research progress**

Wenjing Liu^1^, Yujia Cui^1^, Jieya Wei^1^, Jianxun Sun^1^, Liwei Zheng^1^, Jing Xie^1^*

1. State Key Laboratory of Oral Diseases, West China Hospital of Stomatology, Sichuan University, Chengdu, China

Jing Xie, PH.D.

Professor of State Key Laboratory of Oral Diseases,

West China Hospital of Stomatology,

Sichuan University, Chengdu Sichuan 610064, CHINA

Email: [xiejing2012@scu.edu.cn](mailto:xiejing2012@scu.edu.cn)

**Abstract**

Homeostasis depends on the close connection and intimate molecular exchange between extracellular, intracellular and intercellular networks. Intercellular communication is largely mediated by gap junctions (GJs), a type of specialized membrane contact composed of variable number of channels that enable direct communication between cells by allowing small molecules to pass directly into the [cytoplasm](https://www.sciencedirect.com/topics/medicine-and-dentistry/cytoplasm) of neighbouring cells. Although considerable evidence indicates that gap junctions contribute to the functions of many organs, such as the bone, intestine, kidney, heart, brain and nerve, less is known about their role in oral development and disease. In this review, the current progress in understanding the background of connexins and the functions of gap junctions in oral development and diseases is discussed. The homeostasis of tooth and periodontal tissues, normal tooth and maxillofacial development, saliva secretion and the integrity of the oral mucosa depend on the proper function of gap junctions. Knowledge of this pattern of cell-cell communication is required for a better understanding of oral diseases. With the ever-increasing understanding of connexins in oral diseases, therapeutic strategies could be developed to target these membrane channels in various oral diseases and maxillofacial dysplasia.

**Keywords:** Gap junction, Connexin, Oral disease, Oral health, Tooth, Periodontal, Salivary Gland, Oral Cancer.

**1. Introduction**

Cell-cell communication is vital for cell differentiation, morphogenesis, cell growth and homeostasis in multicellular organisms. ^1^ It was described as “the music that the nucleus hears”, and once it is dissonant, abnormal communication between cells may disrupt biological processes. ^2^ The indispensable components of cell-cell communication include tight junctions, anchoring junctions (adherens, desmosomes, focal adhesions and hemidesmosomes) and communication junctions (gap junctions, pannexins, ion channels, and chemical synapses). ^1^ Gap junctions are clusters of intercellular channels facilitating a direct connection between the cytoplasm of two neighbouring cells to mediate intercellular communication. ^3^ These channels are formed by channel-forming proteins that are densely packed into spatial microdomains of the plasma membrane. Three families of channel forming proteins have been identified, i.e., innexins, connexins and pannexins, among which innexins are located in the protostome and the other two families are present in deuterostomes. The pannexin family is considered a special type of channel-forming proteins that functions as a hemichannel. ^4^ The connexin family exists in the form of hemichannels and assembles into gap junctions in vertebrates. In 1952, Weidman described gap junctions in the myocardium, and thereafter Furshpan and Potter detected these in neurons. ^5, 6^ Currently, 21 connexins have been identified in humans and 20 have been detected in the mouse genome. Gap junctions play pivotal roles in a wide range of physiological processes, including electrical activation of the heart, ^7^ neuronal signalling, ^8^ hormone secretion, ^9^ auditory function, ^10^ wound healing, ^11^ immune functions, ^12^ inflammatory disorders^13^ and bone remodelling. ^14^ Moreover, gap junctions promote the brain metastasis of carcinoma into astrocytes through cGAMP transfer. ^15^ The oral cavity and its appendices are exposed to an intricate environment and considerable mechanical stress. ^16^ Tight polar connections between epithelial cells, which protect the oral mucosa from microbial infections and mechanical stress, are generated from various cell-cell and cell-extracellular matrix junctions.^17^ However, compared with the well-discussed roles in physiological processes, the functions of gap junctions in oral tissues under healthy and pathological conditions remain to be further explored. Thus, in this review, we have focused on the roles of gap junctions in oral development and oral diseases.

**2. Gap junctions formed by connexins**

All junctional channels have an analogous integral structure. However, unlike other membrane channels, different gene families encode the membrane proteins that form junctional channels in different animal phyla. ^18^ In vertebrates, the corresponding genes are denominated with a symbol beginning with “*GJ*” (for gap junction), which represents a virtual narrow separation of 2-4 nm between two neighbouring cells observed using a transmission electron microscope (TEM). ^19^ Meanwhile, the proteins are generally referred to with an abbreviation beginning with “Cx” (for connexin) combined with a number corresponding to the approximate molecular mass of the predicted polypeptide in kilodaltons (kDa). ^20^

**2.1 Structure and function of the connexin hemichannel**

**2.1.1 Structure of connexins**

Each connexin protein is composed of four transmembrane α-helices (TM1-TM4) connected by two extracellular loops and one intracellular loop. ^21^ Their long C-termini (CT) and short N-termini (NT) are located on the cytoplasmic side of the membrane (Fig. 1A). Three cysteine residues reside in each of the two extracellular loops and a proline residue is strictly conserved in connexin proteins, which are critical for intramolecular stabilization. ^18, 22^ Currently, connexins are categorized into five subfamilies, i.e., α, β, γ, δ, and ε or GJA, GJB, GJC, GJD, and GJE, according to the differences and similarities in the amino acid sequences. ^23^ The distribution of connexins (Cxs) varies according to the cell types, the developmental period and the species, partially due to their trafficking towards the plasma membrane after synthesis in the endoplasmic reticulum. Connexin43 (Cx43) is also expressed on mitochondrial membranes and is called mitochondrial Cx43 (mito Cx43). ^24^ As shown in our previous study, Cx43 localizes in the cytoplasm and dendritic processes of osteocytes and even clusters as plaques at the intersection of the dendritic processes of two cells (Fig. 2A). ^25^ We also observed a scattered, punctate distribution of Cx43 at the sites of cell-cell contacts in osteoblast (Fig. 2B).

**2.1.2 Connexin phosphorylation**

The connexin “lifecycle” is a complex process involving the transcription of a specific connexin gene, trafficking, assembly, synthesis and turnover. Several phosphorylation events at multiple sites on connexins have been linked to GJ internalization and turnover. Functional data on phosphorylation has been reported for Cx32, Cx43, Cx45 and Cx56. The C-terminal region of the connexin proteins appears to be the main region that is phosphorylated, except for Cx56, which also contains phosphorylation sites within the cytoplasmic loop region, in addition to its C-terminal domain. ^26,27^ No reports have identified phosphorylation sites at the N-terminus of connexins. Cx26 is the only connexin that exists in a non-phosphorylated state. ^28^ Notably, connexins have rapid turnover rates as membrane proteins. For instance, the half-life of Cx32 in rodent hepatocytes is less than 5 hours in vivo. ^29^ The phosphorylation of Connexin43 (Cx43) at different sites also controls gap junction degradation. ^30^ According to Fernandes R *et al.*, Cx43 phosphorylation attenuates the assembly of gap junctions and potentially leads to Cx43 degradation. ^31^ Moreover, connexin phosphorylation is also associated with the gating of hemichannels or intact gap junction channels. When Cx43 is phosphorylated at the MAPK sites in the presence of a normal extracellular [Ca^2+^] concentration, connexin hemichannels are closed. However, dephosphorylation of those sites by phosphatases in response to a biological stressor, such as hyperosmolarity, induces opening of the hemichannels, resulting in the influx of extracellular ions that impair the function of cells. ^32^ Phosphorylation of Cxs is very important in regulating gap junctional intercellular communication (GJIC), several other sites have been identified and additional phosphorylation sites will likely be identified in the future.

**2.2 Gap junctions**

**2.2.1 Structure of gap junctions**

Six connexin subunits are arranged into a hexamer, forming an annular torus structure around an aqueous pore, which is called a hemichannel or a connexon. Connexons are classified into homomeric (composed of a single connexin protein) and heteromeric (composed of two or more different connexins) channels based on the composition of the channel. Those connexons are then transported to the cell membrane surface, where they dock with a partner connexon from an adjacent cell, forming an intercellular channel that spans the two cells and is called a gap junction channel. ^33^ In addition to homomeric, homotypic channels, a diverse arrangement of gap junctional channels exists between apposed cells. The gap junction channel is classified as homotypic when it is formed from connexons with the same composition. Conversely, if the components of the connexons differ, the gap junctional channel is defined as heterotypic (Fig. 1B). ^34^

**2.2.2 Function of gap junctions**

Gap junctions display a relatively low substrate specificity and are permeable to a wide variety of molecules with mass less than 1 kDa, such as small metabolites, ions and intracellular signalling molecules (i.e., various ions, ATP, ADP, cAMP, amino acids, small peptides, glucose, inositol triphosphate, cyclic nucleotides, and oligonucleotides). ^35^ Gap junctional intercellular communication is actively involved in virtually all aspects of the cellular life cycle, ranging from cell growth, differentiation, and function to cell death. The transfer of current and electrical coupling between cells are key factors regulating the function of excitable tissues, e.g., the heart, in which rapid current transmission is mediated by gap junctional channels between adjacent cells. ^36^ In addition to the heart, gap junctions provide a direct pathway of low resistance for the spread of presynaptic electrical currents to the postsynaptic site in electrical synapses of neurons. ^37^ Moreover, gap junctions facilitate the diffusion of signals from various molecules. Based on accumulating evidence, some Cx channels are permeable to certain soluble second messengers, amino acids, nucleotides and glucose and its metabolites. ^38^ Furthermore, cell-to-cell propagation of calcium waves, which may be initiated by a focal mechanical, electrical or hormonal stimulus, serve to coordinate a global cellular response by diffusing IP_3_ through the gap junctions between cells. ^39^

**2.2.3 Regulatory effects of growth factors on gap junctions**

Given the diversity of gap junction structures, the mechanisms controlling gap junction activity are complex. Various factors are involved in the regulation of GJIC, e.g., growth factors and changes in extracellular matrix. ^40, 41^ Several growth factors have been confirmed to be involved in the regulation of GJIC, and different growth factors induce distinct and even opposite effects on gap junctions.

The effect of epidermal growth factor (EGF) on GJIC of different cell types has been reported. In most cases, EGF reduced the intercellular communication *via* GJs. For instance, the application of 10 ng/ml epidermal growth factor (EGF) for 24 h reduced GJIC in human keratinocytes, and this inhibitory effect of EGF was induced by the MAPK-mediated phosphorylation of Cx43 at Ser255. ^42^ In addition, EGF decreased the expression of the Cx43 protein in cultured rat cortical astrocytes. ^43^ However, in some cell types, such as the K7 human kidney epithelial cell line and granulosa cells, EGF increased the amount of the Cx43 transcript and protein, as well as the function of GJs. ^44,45^ In addition to affecting the functional state of Cx43-mediated GJs, EGF has been reported to modulate other Cx proteins, such as Cx32, which is upregulated by EGF in hepatocytes. ^46^

Controversies exist regarding the effect of [platelet-derived growth factor](https://www.sciencedirect.com/topics/biochemistry-genetics-and-molecular-biology/platelet-derived-growth-factor) (PDGF) on GJs. The addition of PDGF to mesangial cell cultures causes a rapid and transient inhibition of GJIC, with maximal inhibition occurring 15 minutes after PDGF exposure and returning to control levels after 90 minutes. ^47^After the transfection of T51B cells, a rat liver epithelial cell line lacking endogenous PDGF receptors, with a retrovirus encoding wild type human PDGF receptor, GJIC was completely and transiently interrupted upon treatment with PDGF. ^48^ This induced change was associated with increased Cx43 phosphorylation and MAPK activation. PDGF does not affect GJIC in cells transfected with Cx43 truncated at amino acid 256 and carrying a myc tag appended to its C-terminus, suggesting that a Cx43 target site is related to the reduction in GJIC. ^49^ However, PDGF was recently shown to increase Cx43 expression under hypoxic conditions. ^50^

Similar to PDGF, fibroblast growth factor-2 (FGF-2) was shown to exert a biphasic effect on GJIC in cardiac fibroblasts, reducing it within 30 min (short-term effect), but increasing it after longer periods (> 6 h).^51^ FGF-2 also reduced the levels of the Cx43 transcript and protein in astroglial cells.^52^ This FGF-2-induced reduction was likely due to the PKC-mediated phosphorylation of Cx43 at serine 368. ^53^ In cardiomyocytes, FGF signalling was shown to be essential for Cx43 phosphorylation and cardiac gap junction maintenance. ^54^ FGF-5 and FGF-9 induce a decrease in the number of gap junctions in specific brain regions. ^55^ As shown in our previous study, FGF-7 induces the expression of Cx43 and enhances the function of GJs in osteocytes. ^56^ Thus, members of the fibroblast growth factor family affect GJIC in a complex manner.

According to our recent studies, transforming growth factor-beta 1 (TGF-β1) also enhances GJIC by upregulating the level of the Cx43 protein in osteocytes and chondrocytes.^57, 58, 59^ The mechanism by which TGF-β1 increases Cx43 expression is through the activation of the ERK and Smad signalling pathways. ^57^ However, the role of TGF-β1 in modulating the expression of the Cx43 transcript and protein also depends on the cell type. TGF-β1 upregulates Cx43 expression in human granulosa cells and trophoblast cells. ^60,61^ In contrast, TGF-β1 downregulates Cx43 expression in cultured smooth muscle cells from the human detrusor and in rat hepatic stellate cells.^62, 63^ TGF-β2 was recently shown to induce the expression of the Cx43 protein in MSCs. ^64^ TGF-β3 was also shown to [increase gap junctional communication](https://www.ncbi.nlm.nih.gov/pubmed/15961560) among folliculostellate cells. ^65^ Additional studies are required to elucidate the complicated effects of growth factors on GJs in the future.

**2.2.4 The protein interaction partners of connexins**

Based on accumulating evidence obtained in recent years, gap junction proteins do not act as isolated entities in the plasma membrane, but rather interact with a series of partner proteins that link them to the cytoskeleton and to signalling pathways. Multiple proteins have been reported to interact or only colocalize with gap junction proteins. ^66^ Gap junctions have only recently been reported to interact with the actin cytoskeleton. Squecco ^67^ *et al.* observed the colocalization of Cx43 and F-actin in C2C12 cells. Cx43 was also suggested to interact with α-tubulin and β-tubulin. ^68^ Zonula occludens (ZO) proteins, one ubiquitous type of scaffolding protein, may play a general role in the formation and turnover of gap junctions to regulate intercellular communication, since all Cxs identified to date have been reported to interact with ZO proteins. For example, ZO-1 directly tethers connexins to the actin skeleton, ^69^ and ZO-2 also binds to the C-terminus of Cx43. ^70^ However, functional GJs are formed even when the interaction between connexins and ZO-1 is blocked, suggesting that other mechanisms are also involved in plaque formation. ^71^

Gap junctions are formed through cell-cell contact and homophilic cadherin-cadherin interactions. ^72^ Occludin, a protein involved in the formation of tight junctions, interacts with Cx32 in immortalized mouse hepatocytes. ^73^ As shown in the study by Nusrat *et al*., Cx26 interacts with the coiled-coil domain of occludin in epithelial cells. ^74^ Claudins are another component of the tight junction complexes that have a similar topology to Cxs. Claudin-1 colocalizes with Cx32 in rat hepatocytes lines, ^75^ and claudin-5 coprecipitates with Cx43 in porcine blood-brain barrier endothelial cells. ^76^ In addition to the proteins forming tight junctions, N-cadherin, the core component of adherens junctions, also plays a central role in mediating cell-cell interactions. In NIH3T3 cells, Cx43 interacts with an N-cadherin-containing multiprotein complex. Moreover, this interaction has been shown to be essential for gap junction formation. ^77^ When the N-cadherin gene is deleted in N-cadherin knockout mice, cardiomyocytes are deficient gap junctions. ^78^ However, an ongoing controversy exists over the role of N-cadherin in the formation of gap junction. According to [Govindarajan](https://www.ncbi.nlm.nih.gov/pubmed/?term=Govindarajan R%5BAuthor%5D&cauthor=true&cauthor_uid=20881055) e*t al.*, ^79^ the expression of N-cadherin attenuates GJ assembly in rat liver epithelial cells by inducing the endocytosis of Cx43.

Some membrane channels and enzymes are also connexin partners. Sodium channel complexes interact with Cx43 in ventricular myocytes. ^80^ Aquaporin, a member of the major intrinsic protein (MIP) superfamily, interacts with two binding sites within the intracellular loop region of Cx50. ^81^ The long cytosolic C-terminus (CT) of Cx43 is required for the proper function of Cx43 gap junctions. The C-terminus of several connexins (e.g., Cx43) contains consensus phosphorylation sites for several proteins, including Src protein tyrosine kinases, ^82^ Akt ^83^ and [mitogen-activated protein kinase](https://www.sciencedirect.com/topics/biochemistry-genetics-and-molecular-biology/mitogen-activated-protein-kinase) (MAPK). ^84^ Protein kinase C (PKC) displays partial colocalization with Cx43 and directly phosphorylates Cx43 at Ser368, ^85^ thus modulating various phases of the Cx43 life cycle, including gap junction assembly, gap junction channel permeability, and Cx43 endocytosis and degradation. Conceivably, the interactions between Cxs and other proteins have important functions under physiological conditions and in associated diseases, such as oral disease.

**3. Connexins in oral development and oral diseases**

**3.1 Connexins in oral and maxillofacial development**

**3.1.1 Connexins in tooth development**

Tooth development depends on the sequential and reciprocal interactions between the epithelium and mesenchyme. In the developing tooth germ of neonatal rats, Cx43 is distributed both in epithelial and mesenchymal dental cells; ^86^ Cx43 has been detected between ameloblasts and between odontoblasts. ^87,88^ Cx43 expression gradually increases with the progression of odontoblast maturation from pre-odontoblast to old odontoblasts. ^89^ During ameloblast development, Cx43 expression shows a transient decrease in the late presecretory ameloblasts before enamel formation and then increases during the secretory stage. ^90^ At the maturation stage of enamel, GJs contribute to enamel formation by transporting ions from the papillary layer to ameloblasts. ^91^ As shown in the study by Toth *et al.*, ^92^ dominant negative G60S mutants of Cx43 result in ameloblast dysregulation and enamel hypoplasia. Once tooth germ development is completed, consistent expression of Cx43 at high levels in human dental follicle cells (HDFCs) is essential for tooth eruption. ^93^ In addition to the involvement of Cx43 in enamel development, Cx32 has also been detected in the developing enamel organ. ^94^ Based on these findings, Cxs are involved in tooth development, and a certain connexin may have distinct roles in odontogenesis and tooth homeostasis.

**3.1.2 Connexins in maxillofacial development**

Cx43 plays a critical role in the development of maxillofacial structures. Patients who suffer from oculodentodigital dysplasia *(ODDD)*, which is caused by mutations in *Cx43/GJα1*, present oral dysfunction, including oral clefting, numerous cavities and tooth loss. ^95^ Cx43 and Cx32 are related to differentiation and growth during the early phase of submandibular gland development. During this period, Cx43 is considered to contribute to the branching morphogenesis ^96^ and the contractile function of myoepithelial cells, while Cx32 expression may correspond to an increase and decrease in the number of proacinar and mature acinar cells, respectively. ^97^ Cx43 also plays an important role in the initiation of papillary pattern formation and morphogenesis in the tongue. ^98^ For instance, Cx43 regulates the localization of keratins to pre-pattern of the bud region during the formation of the circumvallate papilla. ^99^ Compared with the available data on the functions of Cxs in tooth development, much less is known about the roles of Cxs and GJs in maxillofacial development, such as palate and mandible development.

**3.2 Connexins in oral diseases**

**3.2.1 Connexins in periodontal tissue**

The periodontal ligament (PDL) is a soft connective tissue that resides between the alveolar bone and tooth to sustain teeth and preserve tissue homeostasis. Periodontal tissue homeostasis depends on a complicated cellular network that transmits signals between periodontal ligament fibroblasts (PDLFs). ^100^ Cx proteins (Cx32, Cx40, Cx43, and Cx45) are expressed in PDLFs, ^101,102^ while Cx26 and Cx37 have not been detected. Those proteins have two main functions. One is the correlation between Cx32 and the secretory function of PDLFs, i.e., the ability to produce type I and type III collagen and fibronectin. On the other hand, Cx40 and Cx45 are considered to relate to the contractile function of PDLFs, which may relate to tooth eruption. ^103^ Moreover, Cx43 is involved in the transmission of signals from mechanical stimuli in the PDL. During orthodontic tooth movement, Cx43 expression is increased in rat PDLFs. ^104^ Mechanical tension also increases the expression of Cx43 and promotes GJIC in a time-dependent manner, while 24 h cyclic stretches downregulate the expression of Cx43 in the membrane of hPDLFs. ^102,105^ Cxs have also been studied in human gingival epithelium, where keratinocytes normally express Cx26 and Cx43 at high levels, ^106-108^ and Cx43 even forms plaques in fibroblasts. In the rat gingival epithelia, Cx43 was detected in the basal layer and middle of the prickle cell layer. Interestingly, in the human gingival epithelia, Cx43 expression showed a progressive decrease from the spinous layers of the oral gingival epithelium to the sulcular epithelium and parts of the junctional epithelium. ^106,107^ Cx26 was detected in the granular cell layer and lower part of the squamous cell layer. ^109^ Nevertheless, Cx43 was downregulated at the early stage of gingival wound healing, which may contribute to the fast wound healing of the gingival tissue. ^110^ However, to the best of our knowledge, no information is available on the role of gap junctions in periodontal disease.

**3.2.2 Connexins in tooth development and diseases**

Dental pulp contains fibroblasts, odontoblasts and undifferentiated mesenchymal cells, and dental pulp fibroblasts (DPFs) are major components among these cells. ^111^ Cx32 and Cx43 expression have been observed in human DPFs. In cultured human pulp cells, Cx43 expression is upregulated during mineralization processes, indicating that Cx43 might play a role in mineralization. ^86^ This finding was further supported by a study showing that the inhibition of Cx43 attenuates the odontoblastic differentiation and mineralization of rat dental pulp cells. ^112^ In addition, Shiting *et al*. ^113^ found that the overexpression of Cx43 potentiated the odontoblastic differentiation of dental pulp stem cells (DPSCs), suggesting that Cx43 is involved in the differentiation of DPSCs into odontoblasts. Cx43 is a marker of the viability of dental pulp tissue, as Cx43 expression is reduced in aged human dental pulp. A reduction in Cx43 expression may be one characteristic of aged pulp. ^114^ This hypothesis was further verified by the rapid degradation of Cx43 in pulp cells exposed to physical stimuli such as heat. ^115^ In intact adult teeth, Cx43 forms gap junctions between odontoblasts.^116^ Microscopic observations indicate the localization of gap junctions between the bodies of odontoblasts and between the bodies and processes of odontoblasts. ^117^ Similarly, Cx26 is expressed at low levels in the odontoblast layer, while Cx32 is not detected in odontoblasts. ^118^ In carious teeth, Cx43 expression is upregulated in mature odontoblasts in the vicinity of carious lesions, combined with the prominent expression of Cx43 in the interodontoblastic cells. ^119^ However, the expression of Cx43 between odontoblasts is reduced in reactionary dentin that forms during dentin caries development. ^120^ Notably, fluoride, which is recognized to protect against tooth decay, increases Cx43 and Cx32 expression, but decreases Cx45 expression in rat incisor pulp. ^121^ Nevertheless, the scientiﬁc evidence supporting the correlation between Cxs and caries is insufficient, and the mechanism by which fluoride regulates connexin expression in odontoblasts remains to be elucidated.

**3.2.3 Connexins in oral cancer**

Connexins may be associated with cell growth, since the absence of GJIC can result in an accumulation of growth factors in cells ^122^ and a suppression of contact inhibition, which together lead to cell proliferation. ^123^ Additionally, a decrease in Cx43 and Cx32 expression may result in the uncontrolled proliferation and abnormal differentiation of various benign and malignant tumour cells. ^124-126^ Oral squamous cell carcinoma (OSCC) is the most prevalent and most commonly studied oral cancer. ^127^ However, the potential role of the oral microbiome in OSCC has not been clearly elucidated. In the dysplasia-free oral mucosa, Cx43 is mainly expressed on the membrane in the stratum spinosum and stratum granulosum, but is not expressed in the stratum corneum. However, a significant increase in cytoplasmic Cx43 expression and a loss of GJIC have been observed during the early carcinogenesis of OSCC. Therefore, membrane Cx43 levels might be an independent biomarker for early changes associated with oral squamous cell carcinoma. ^128^ Moreover, Cx43^+^ fibroblasts are enriched in the stroma of OSCC, which may be a hallmark for judging oral SCC invasion. ^129^ Changes in Cx43 expression have been detected in malignant and benign tumours. In another study, the downregulation of Cx43 and Cx32 expression was observed in keratocystic odontogenic tumours, one of the most frequently occurring types of benign odontogenic tumours. ^130^ In addition to studies of changes in Cx43 and Cx32 expression during carcinogenesis, Cx26 expression was also reported to be reduced in tongue carcinoma. ^131,132^ Recently, many studies have focused on therapies that restore connexin-mediated GJIC in oral cancer. For example, all-trans retinoic acid was shown to be beneficial for OSCC cells to regain cell-cell communication by increasing Cx32 and Cx43 expression. ^133^ According to Marie *et al*., gap junctional intercellular communication is enhanced by docetaxel in salivary gland carcinoma, concomitant with an increase in Cx43 expression and its membrane localization. ^134^ Lycopene significantly increased Cx43 expression and GJIC between KB-1 cells, which originate from a human oral cavity tumour. ^135^ Based on the information described above, Cx43 may be a tumour suppressor and a potentially novel therapeutic target for oral cancer.

**3.2.4 Connexins in diseases caused by gene mutations**

Functional studies have begun to identify some of the underlying mechanisms by which connexin channel mutations contribute to oral cavity diseases. Keratitis-ichthyosis-deafness (KID) syndrome is a rare ectodermal dysplasia caused by mutations in the *GJB2* gene, which is responsible for the production of the Cx26 protein, a protein present in the epithelial gap junctions that is postulated to be associated with the differentiation of ectodermally derived tissues. Phenotypic features associated with Cx26 mutations are significant visual and auditory impairments. Affected patients are also at increased risk of developing epithelial malignancies. One KID syndrome has been noted to confer a predisposition to SCC. ^136^ Approximately 11% of patients with KID syndrome develop this condition. ^137^ Oculodentodigital dysplasia (ODDD) is another congenital disorder caused by a mutation in the *GJα1* gene, which encodes Cx43. ^138^ It is characterized by multiple phenotypic abnormalities involving the face, limbs, teeth, and eyes, as well as neurological symptomatology. Concerning the teeth, microdontia is present in one-fifth of the patients. More frequently, patients suffer from the amelogenesis imperfecta (AI), hypoplastic type. Other dental symptoms reported include malocclusion, delayed tooth development, pulp stones, tooth loss and missing teeth.^139^

**3.2.5 Connexins and wound healing in the oral cavity**

The suppression of Cx43 expression or function promotes skin wound healing and alleviates scarring. Corresponding to this finding, Cx43 expression is substantially decreased in human gingival fibroblasts at the early stage of wound closure, and Cx43 regulates the expression of wound-healing genes in gingiva. Thus, downregulation of Cx43 appears to be conductive to fast and scarless wound healing in gingival tissues. ^110,140,141^ Consistent with this finding, Masato *et al.* also observed a wound-induced decrease and subsequent increase in Cx43 expression in the hamster tongue epithelium. ^131^ Interestingly, in contrast to skin after injury, the expression of Cx43 in buccal mucosa wounds decreases and remains at a low level for 14 days. Increased Cx43 expression affects MMP-1 synthesis, which facilitates scar formation. ^142^ Together, these studies explain why the oral mucosa is less prone to form a scar after wound healing compared to skin.

**3.2.6 Connexins in** **the salivary gland**

Salivary glands play an important role in oral biology by secreting saliva to provide water for lubrication, as well as electrolytes, mucus, enzymes and antibacterial compounds. Abnormal function of the salivary gland can lead to an extensive deterioration of oral health. Gap junctions have recently been suggested to be involved in maintaining salivary gland function. ^143^ Cx26 and Cx32 colocalize within the same gap junctional plaque between acinar cells in rat parotid glands, but no expression of these two proteins is observed in the ducts. ^144,145^ In rat submandibular and sublingual glands, Cx32 is distributed at the membranes between acinar cells and Cx43 is localized at the gap junctions between the thin processes of myoepithelial cells, suggesting that Cx32-meditated GJs are related to regulation of the secretory function of acinar cells and Cx43-mediated GJs are involved in the contractile function of myoepithelial cells. ^146,147^ However, these studies are still limited to exploring phenotypes, and the role of gap junctions in specific salivary gland diseases is unknown. Therefore, an analysis of connexins and gap junctions will hopefully contribute to the study of salivary diseases.

**4.** **Conclusions and perspectives**

As data documenting the functions of [connexins](https://www.sciencedirect.com/topics/medicine-and-dentistry/gap-junction-protein) in the oral health and oral disease are still limited, information is mainly confined to the distribution of Cxs in diverse oral tissues during different developmental phases. Far fewer reports have described the role of functional GJs in oral diseases such as periodontitis and chronic apical periodontitis. Previous studies have provided support for additional roles of Cxs in oral development and the pathogenesis and prognosis of oral diseases. Studies of various Cxs in oral tissues are categorized in Table 1. Collectively, Cxs and GJs play important roles in maintaining the normal development and function of oral tissues. Specific Cxs may potentially represent molecular targets for the treatment of certain oral diseases. Therefore, Cxs and gap junctions appear to be a very interesting field for additional research.

**ACKNOWLEDGEMENTS**

This study was supported by the National Natural Science Foundation of China (81600840, 81771047 to Jing Xie).

**AUTHOR CONTRIBUTIONS**

J. X. designed the concept. W.J.L. drafted the manuscript. Y.J.C., J.Y.W., J.X.S. and L.W.Z. revised the manuscript. All authors reviewed the manuscript and J.X. made a final approval.

**ADDITIONAL INFORMATION**

**Competing interests:** The authors declare no competing interests.

**REFERENCES:**

1. Brucher, B. L. & Jamall, I. S. Cell-cell communication in the tumor microenvironment, carcinogenesis, and anticancer treatment. *Cell*. *Physiol. Biochem*. **34**, 213-243 (2014).
2. McCrea, P.D., Gu, D. & Balda, M.S. Junctional music that the nucleus hears: cell-cell contact signaling and the modulation of gene activity. [*Cold Spring Harb Perspect Biol*.](https://www.ncbi.nlm.nih.gov/pubmed/?term=Junctional+music+that+the+nucleus+hears%3A+cell-cell+contact+signaling+and+the+modulation+of+gene+activity.) **1**, a002923 (2009).
3. Wiener, J., Spiro, D. & Loewenstein, W. R. Studies on an epithelial (gland) cell junction. II. Surface structure. [*J. Cell Biol*.](https://www.ncbi.nlm.nih.gov/pubmed/?term=STUDIES+ON+AN+EPITHELIAL+(GLAND)+CELL+JUNCTION.+II.+SURFACE+STRUCTURE.) **22**, 587-598 (1964).
4. [Barbe, M.T](https://www.ncbi.nlm.nih.gov/pubmed/?term=Barbe MT%5BAuthor%5D&cauthor=true&cauthor_uid=16565476)., [Monyer, H](https://www.ncbi.nlm.nih.gov/pubmed/?term=Monyer H%5BAuthor%5D&cauthor=true&cauthor_uid=16565476). & [Bruzzone, R](https://www.ncbi.nlm.nih.gov/pubmed/?term=Bruzzone R%5BAuthor%5D&cauthor=true&cauthor_uid=16565476). Cell-Cell Communication Beyond Connexins: The Pannexin Channels. [*Physiology (Bethesda)*.](https://www.ncbi.nlm.nih.gov/pubmed/?term=Cell-Cell+Communication+Beyond+Connexins++The+Pannexin+Channels) **21**, 103-114 (2006).
5. [Weidmann S](https://www.ncbi.nlm.nih.gov/pubmed/?term=WEIDMANN S%5BAuthor%5D&cauthor=true&cauthor_uid=13000763). The electrical constants of Purkinje fibres. *J. Physiol*. **118**, 348-360 (1952) .
6. Furshpan, E. J. & Potter, D. D. Mechanism of nerve-impulse transmission at a crayfish synapse. [*Nature*.](https://www.ncbi.nlm.nih.gov/pubmed/?term=Mechanism+of+nerve-impulse+transmission+at+a+crayfish+synapse.) **17**, 342-343 (1957).
7. Epifantseva, I. & Shaw, R. M. Intracellular trafficking pathways of Cx43 gap junction channels. *Biochim. Biophys. Acta. Biomembr*. **1860**, 40-47 (2018).
8. Spray, D. C. et al. Gap junction mediated signaling between satellite glia and neurons in trigeminal ganglia. *Glia*. **67**, 791-801 (2019).
9. Meda, P. Gap junction proteins are key drivers of endocrine function. *Biochimica Biophysica. Acta. Biomembranes*. **1860**, 124-140 (2018).
10. Jagger, D. J. & Forge, A. Connexins and gap junctions in the inner ear--it's not just about K⁺ recycling. *Cell Tissue Re*s. **360**, 633-644 (2015).
11. Laird, D. W. & Lampe, P. D. Therapeutic strategies targeting connexins. *Nat. Rev. Drug Discov.* **17**, 905-921 (2018).
12. Neijssen, J., Pang, B. & Neefjes, J. Gap junction-mediated intercellular communication in the immune system. *Prog. Biophys. Mol Biol*. **94**, 207-218 (2007).
13. [Wong, P](https://www.ncbi.nlm.nih.gov/pubmed/?term=Wong P%5BAuthor%5D&cauthor=true&cauthor_uid=28098880)., [Laxton, V](https://www.ncbi.nlm.nih.gov/pubmed/?term=Laxton V%5BAuthor%5D&cauthor=true&cauthor_uid=28098880)., [Srivastava, S](https://www.ncbi.nlm.nih.gov/pubmed/?term=Srivastava S%5BAuthor%5D&cauthor=true&cauthor_uid=28098880)., [Chan, Y. W](https://www.ncbi.nlm.nih.gov/pubmed/?term=Chan YW%5BAuthor%5D&cauthor=true&cauthor_uid=28098880). & [Tse, G](https://www.ncbi.nlm.nih.gov/pubmed/?term=Tse G%5BAuthor%5D&cauthor=true&cauthor_uid=28098880). The role of gap junctions in inflammatory and neoplastic disorders (Review). [*Int. J. Mol. Med.*](https://www.ncbi.nlm.nih.gov/pubmed/28098880) **39,** 498-506 (2017).
14. Moorer, M. C. & Stains, J. P. Connexin43 and the Intercellular Signaling Network Regulating Skeletal Remodeling. [*Curr. Osteoporos. Rep*.](https://www.ncbi.nlm.nih.gov/pubmed/?term=Connexin43+and+the+Intercellular+Signaling+Network+Regulating+Skeletal+Remodeling) **15**, 24-31(2017).
15. [Chen, Q](https://www.ncbi.nlm.nih.gov/pubmed/?term=Chen Q%5BAuthor%5D&cauthor=true&cauthor_uid=27225120). et al. Carcinoma-astrocyte gap junctions promote brain metastasis by cGAMP transfer. *Nature.* **533.** 493-498 (2016).
16. [Samiei, M](https://www.ncbi.nlm.nih.gov/pubmed/?term=Samiei M%5BAuthor%5D&cauthor=true&cauthor_uid=31338005). et al. Cell junctions and oral health. [*EXCLI J.*](https://www.ncbi.nlm.nih.gov/pubmed/31338005) **18,** 317-330 (2019).
17. Groeger, S. E. & Meyle, J. Epithelial barrier and oral bacterial infection. *Periodontology*. **69,** 46-67 (2015).
18. Hervé, J. C. & Derangeon, M. Gap-junction-mediated cell-to-cell communication. *Cell Tissue Res*. **352**, 21-31 (2013).
19. [Roy, S](https://www.ncbi.nlm.nih.gov/pubmed/?term=Roy S%5BAuthor%5D&cauthor=true&cauthor_uid=28602949)., [Jiang, J. X](https://www.ncbi.nlm.nih.gov/pubmed/?term=Jiang JX%5BAuthor%5D&cauthor=true&cauthor_uid=28602949) ., [Li, A. F](https://www.ncbi.nlm.nih.gov/pubmed/?term=Li AF%5BAuthor%5D&cauthor=true&cauthor_uid=28602949). & [Kim, D](https://www.ncbi.nlm.nih.gov/pubmed/?term=Kim D%5BAuthor%5D&cauthor=true&cauthor_uid=28602949). Connexin channel and its role in diabetic retinopathy. [*Prog. Retin. Eye. Res.*](https://www.ncbi.nlm.nih.gov/pubmed/28602949) **61**, 35-59 (2017).
20. Beyer, E. C. & Berthoud, M. Gap junction gene and protein families: Connexins, innexins, and pannexins. *Biochim. Biophys. Acta. Biomembr*. **1860**, 5-8 (2018).
21. [Meşe, G](https://www.ncbi.nlm.nih.gov/pubmed/?term=Meşe G%5BAuthor%5D&cauthor=true&cauthor_uid=17934503)., [Richard, G](https://www.ncbi.nlm.nih.gov/pubmed/?term=Richard G%5BAuthor%5D&cauthor=true&cauthor_uid=17934503). & [White, T. W](https://www.ncbi.nlm.nih.gov/pubmed/?term=White TW%5BAuthor%5D&cauthor=true&cauthor_uid=17934503). Gap Junctions: Basic Structure and Function. [*J. Invest. Dermatol.*](https://www.ncbi.nlm.nih.gov/pubmed/17934503) **127**, 2516-2524 (2007).
22. [Krutovskikh, V](https://www.ncbi.nlm.nih.gov/pubmed/?term=Krutovskikh V%5BAuthor%5D&cauthor=true&cauthor_uid=10767631). & [Yamasaki , H](https://www.ncbi.nlm.nih.gov/pubmed/?term=Yamasaki H%5BAuthor%5D&cauthor=true&cauthor_uid=10767631). Connexin gene mutations in human genetic diseases. [*Mutat. Res*.](https://www.ncbi.nlm.nih.gov/pubmed/10767631) **462**, 197-207 (2000).
23. [Beyer, E. C](https://www.ncbi.nlm.nih.gov/pubmed/?term=Beyer EC%5BAuthor%5D&cauthor=true&cauthor_uid=28559187). & [Berthoud,V. M](https://www.ncbi.nlm.nih.gov/pubmed/?term=Berthoud VM%5BAuthor%5D&cauthor=true&cauthor_uid=28559187). Gap junction gene and protein families: Connexins, innexins, and pannexins. [*Biochim. Biophys. Acta. Biomembr*.](https://www.ncbi.nlm.nih.gov/pubmed/28559187) **1860**, 5-8 (2018).
24. Rodríguez-Sinovas, A., Ruiz-Meana, M., Denuc, A. & García-Dorado, D. [Mitochondrial Cx43, an important component of cardiac preconditioning.](https://www.ncbi.nlm.nih.gov/pubmed/28642043) *Biochim Biophys. Acta. Biomembr*. **1860**, 174-181 (2018).
25. [Liu, W](https://www.ncbi.nlm.nih.gov/pubmed/?term=Liu W%5BAuthor%5D&cauthor=true&cauthor_uid=30482881). et al. Transforming growth factor-beta1 up-regulates connexin43 expression in osteocytes via canonical Smad-dependent signaling pathway. [*Biosci. Rep* (in the press).](https://www.ncbi.nlm.nih.gov/pubmed/?term=Transforming+growth+factor-beta1+up-regulates+connexin43+expression+in+osteocytes+via+canonical+Smad-dependent+signaling+pathway.)
26. [Lampe, P. D](https://www.ncbi.nlm.nih.gov/pubmed/?term=Lampe PD%5BAuthor%5D&cauthor=true&cauthor_uid=15109565). & [Lau, A. F](https://www.ncbi.nlm.nih.gov/pubmed/?term=Lau AF%5BAuthor%5D&cauthor=true&cauthor_uid=15109565). The effects of connexin phosphorylation on gap junctional communication. [*Int.J. Biochem. Cell Biol*.](https://www.ncbi.nlm.nih.gov/pubmed/15109565) **36**, 1171-1186 (2004).
27. [Berthoud, V. M](https://www.ncbi.nlm.nih.gov/pubmed/?term=Berthoud VM%5BAuthor%5D&cauthor=true&cauthor_uid=9063450)., [Beyer, E. C](https://www.ncbi.nlm.nih.gov/pubmed/?term=Beyer EC%5BAuthor%5D&cauthor=true&cauthor_uid=9063450), [Kurata, W. E](https://www.ncbi.nlm.nih.gov/pubmed/?term=Kurata WE%5BAuthor%5D&cauthor=true&cauthor_uid=9063450)., [Lau, A. F](https://www.ncbi.nlm.nih.gov/pubmed/?term=Lau AF%5BAuthor%5D&cauthor=true&cauthor_uid=9063450). & [Lampe, P. D](https://www.ncbi.nlm.nih.gov/pubmed/?term=Lampe PD%5BAuthor%5D&cauthor=true&cauthor_uid=9063450). The gap-junction protein connexin 56 is phosphorylated in the intracellular loop and the carboxy-terminal region. [*Eur. J. Biochem*.](https://www.ncbi.nlm.nih.gov/pubmed/?term=The+gap-junction+protein+connexin+56+is+phosphorylated+in+the+intracellular+loop+and+the+carboxy-terminal+region.) **244**, 89-97(1997).
28. [Traub, O](https://www.ncbi.nlm.nih.gov/pubmed/?term=Traub O%5BAuthor%5D&cauthor=true&cauthor_uid=2537831)., [Look, J](https://www.ncbi.nlm.nih.gov/pubmed/?term=Look J%5BAuthor%5D&cauthor=true&cauthor_uid=2537831). & [Dermietzel, R](https://www.ncbi.nlm.nih.gov/pubmed/?term=Dermietzel R%5BAuthor%5D&cauthor=true&cauthor_uid=2537831). Comparative characterization of the 21-kD and 26-kD gap junction proteins in murine liver and cultured hepatocytes. [*J. Cell. Biol.*](https://www.ncbi.nlm.nih.gov/pubmed/?term=Comparative+characterization+of+the+21-kD+and+26-kD+gap+junction+proteins+in+murine+liver+and+cultured+hepatocytes.)**108**, 1039-1051(1989).
29. Goodenough, D. A. & Paul, D. L. [Gap junctions.](https://www.ncbi.nlm.nih.gov/pubmed/20066080) *Cold Spring Harb Perspect Biol*. **1**, a002576 (2009).
30. Solan, J. L. & Lampe, P. D. [Specific Cx43 phosphorylation events regulate gap junction turnover in vivo.](https://www.ncbi.nlm.nih.gov/pubmed/24508467) *FEBS. Lett*. **588**, 1423-1429 (2014).
31. [Fernandes, R](https://www.ncbi.nlm.nih.gov/pubmed/?term=Fernandes R%5BAuthor%5D&cauthor=true&cauthor_uid=15123628)., [Girão, H](https://www.ncbi.nlm.nih.gov/pubmed/?term=Girão H%5BAuthor%5D&cauthor=true&cauthor_uid=15123628). & [Pereira, P](https://www.ncbi.nlm.nih.gov/pubmed/?term=Pereira P%5BAuthor%5D&cauthor=true&cauthor_uid=15123628). High glucose down-regulates intercellular communication in retinal endothelial cells by enhancing degradation of connexin 43 by a proteasome-dependent mechanism. [*J. Biol.Chem*.](https://www.ncbi.nlm.nih.gov/pubmed/?term=High+glucose+down-regulates+intercellular+communication+in+retinal+endothelial+cells+by+enhancing+degradation+of+connexin+43+by+a+proteasome-dependent+mechanism.) **279**, 27219-27224 (2004).
32. John, S., Cesario, D. & Weiss, J. N. [Gap junctional hemichannels in the heart.](https://www.ncbi.nlm.nih.gov/pubmed/12940935) *Acta Physiol. Scand.* **179**, 23-31 (2003).
33. Lilly, E., Sellitto, C., Milstone, L. M., White, T.W. Connexin channels in congenital skin disorders. *Semin.Cell Dev. Biol*. **50**, 4-12 (2016).
34. Evans, W. H. & Martin, P. E. Gap junctions: structure and function. [*Mol. Membr. Biol.*](https://www.ncbi.nlm.nih.gov/pubmed/12126230) **19**, 121-136 (2002).
35. Zappitelli, T. & Aubin, J. E. The "connexin" between bone cells and skeletal functions. *J. Cell. Biochem*. **115**, 1646-1658 (2014).
36. Epifantseva, I. & Shaw, R. M. Intracellular trafficking pathways of Cx43 gap junction channels. *Biochim. Biophys. Acta. Biomembr.* **1860**, 40-47 (2018).
37. [Pereda, A. E](https://www.ncbi.nlm.nih.gov/pubmed/?term=Pereda AE%5BAuthor%5D&cauthor=true&cauthor_uid=22659675)., [Curti, S](https://www.ncbi.nlm.nih.gov/pubmed/?term=Curti S%5BAuthor%5D&cauthor=true&cauthor_uid=22659675)., [Hoge, G](https://www.ncbi.nlm.nih.gov/pubmed/?term=Hoge G%5BAuthor%5D&cauthor=true&cauthor_uid=22659675). et al. Gap junction-mediated electrical transmission: regulatory mechanisms and plasticity. [*Biochim Biophys Acta*.](https://www.ncbi.nlm.nih.gov/pubmed/?term=Gap+junction-mediated+electrical+transmission%3A+regulatory+mechanisms+and+plasticityGap+junction-mediated+electrical+transmission%3A+regulatory+mechanisms+and+plasticity) **1828**, 134-146 (2013).
38. Hervé, J. C. & Derangeon, M. Gap-junction-mediated cell-to-cell communication. *Cell Tissue Res*. **352**, 21-31 (2013).
39. [Dupont, G](https://www.ncbi.nlm.nih.gov/pubmed/?term=Dupont G%5BAuthor%5D&cauthor=true&cauthor_uid=17560283)., [Combettes, L](https://www.ncbi.nlm.nih.gov/pubmed/?term=Combettes L%5BAuthor%5D&cauthor=true&cauthor_uid=17560283). & [Leybaert, L](https://www.ncbi.nlm.nih.gov/pubmed/?term=Leybaert L%5BAuthor%5D&cauthor=true&cauthor_uid=17560283). Calcium dynamics: spatio-temporal organization from the subcellular to the organ level. [*Int. Rev. Cytol*.](https://www.ncbi.nlm.nih.gov/pubmed/?term=Calcium+dynamics%3A+spatio-temporal+organization+from+the+subcellular+to+the+organ+level.) **261**, 193-245 (2007).
40. [Zhang, D](https://www.ncbi.nlm.nih.gov/pubmed/?term=Zhang D%5BAuthor%5D&cauthor=true&cauthor_uid=30476913). et al. Extracellular Matrix Elasticity Regulates Osteocyte Gap Junction Elongation: Involvement of Paxillin in Intracellular Signal Transduction. *Cell. Physiol. Biochem*. **51**, 1013-1026 (2018).
41. Xie, J. et al. Compliant Substratum Changes Osteocyte Functions: The Role of ITGB3/FAK/β-Catenin Signaling Matters. *ACS Applied. Bio. Materials.* **1**, 792-801 (2018).
42. [Kanemitsu, M. Y](https://www.ncbi.nlm.nih.gov/pubmed/?term=Kanemitsu MY%5BAuthor%5D&cauthor=true&cauthor_uid=8241569). & [Lau, A. F](https://www.ncbi.nlm.nih.gov/pubmed/?term=Lau AF%5BAuthor%5D&cauthor=true&cauthor_uid=8241569). Epidermal growth factor stimulates the disruption of gap junctional communication and connexin43 phosphorylation independent of 12-0-tetradecanoylphorbol 13-acetate-sensitive protein kinase C: the possible involvement of mitogen-activated protein kinase. [*Mol. Biol. Cell*.](https://www.ncbi.nlm.nih.gov/pubmed?term=epidermal growth factor stimulated the disruption of gap junctional communication and connexin43 phosphorylation independent of 12-0-tetradecanoylphorbol 13-acetate-sensitive protein kinase c the possible involvement of mitogen-activated protein kinase.&cmd=correctspelling) **4**, 837-848 (1993).
43. [Ueki, T](https://www.ncbi.nlm.nih.gov/pubmed/?term=Ueki T%5BAuthor%5D&cauthor=true&cauthor_uid=11684338). et al. Epidermal growth factor down-regulates connexin-43 expression in cultured rat cortical astrocytes. [*Neurosci Lett.*](https://www.ncbi.nlm.nih.gov/pubmed/11684338) **313**, 53-56 (2001).
44. [Rivedal, E](https://www.ncbi.nlm.nih.gov/pubmed/?term=Rivedal E%5BAuthor%5D&cauthor=true&cauthor_uid=11532878). & [Opsahl, H](https://www.ncbi.nlm.nih.gov/pubmed/?term=Opsahl H%5BAuthor%5D&cauthor=true&cauthor_uid=11532878). Role of PKC and MAP kinase in EGF- and TPA-induced connexin43 phosphorylation and inhibition of gap junction intercellular communication in rat liver epithelial cells. [*Carcinogenesis.*](https://www.ncbi.nlm.nih.gov/pubmed/?term=E.+Rivedal+and+H.+Opsahl%2C+Carcinogenesis%2C) **22**, 1543-1550 (2001).
45. [Kennedy, K.L](https://www.ncbi.nlm.nih.gov/pubmed/?term=Kennedy KL%5BAuthor%5D&cauthor=true&cauthor_uid=12420300)., [Floyd , A. A](https://www.ncbi.nlm.nih.gov/pubmed/?term=Floyd AA%5BAuthor%5D&cauthor=true&cauthor_uid=12420300)., [Clarkson , A. M](https://www.ncbi.nlm.nih.gov/pubmed/?term=Clarkson AM%5BAuthor%5D&cauthor=true&cauthor_uid=12420300). & [Lee, V. H](https://www.ncbi.nlm.nih.gov/pubmed/?term=Lee VH%5BAuthor%5D&cauthor=true&cauthor_uid=12420300). Epidermal growth factor regulation of connexin 43 in cultured granulosa cells from preantral rabbit follicles. [*Mol Reprod Dev*.](https://www.ncbi.nlm.nih.gov/pubmed/12420300) **64,** 61-69 (2003).
46. [Kojima, T](https://www.ncbi.nlm.nih.gov/pubmed/?term=Kojima T%5BAuthor%5D&cauthor=true&cauthor_uid=7615657)., [Mitaka, T](https://www.ncbi.nlm.nih.gov/pubmed/?term=Mitaka T%5BAuthor%5D&cauthor=true&cauthor_uid=7615657)., [Paul, D. L](https://www.ncbi.nlm.nih.gov/pubmed/?term=Paul DL%5BAuthor%5D&cauthor=true&cauthor_uid=7615657)., [Mori, M](https://www.ncbi.nlm.nih.gov/pubmed/?term=Mori M%5BAuthor%5D&cauthor=true&cauthor_uid=7615657). & [Mochizuki, Y](https://www.ncbi.nlm.nih.gov/pubmed/?term=Mochizuki Y%5BAuthor%5D&cauthor=true&cauthor_uid=7615657). Reappearance and long-term maintenance of connexin32 in proliferated adult rat hepatocytes: use of serum-free L-15 medium supplemented with EGF and DMSO. [*J. Cell. Sci.*](https://www.ncbi.nlm.nih.gov/pubmed/7615657) **108**, 1347-1357 (1995).
47. Yao, J., Morioka, T. & Oite T. [PDGF regulates gap junction communication and connexin43 phosphorylation by PI 3-kinase in mesangial cells.](https://www.ncbi.nlm.nih.gov/pubmed/10792610) *Kidney Int.* **57,** 1915-1926 (2000).
48. [Hossain, M. Z](https://www.ncbi.nlm.nih.gov/pubmed/?term=Hossain MZ%5BAuthor%5D&cauthor=true&cauthor_uid=9397157)., [Ao, P](https://www.ncbi.nlm.nih.gov/pubmed/?term=Ao P%5BAuthor%5D&cauthor=true&cauthor_uid=9397157). & [Boynton, A. L](https://www.ncbi.nlm.nih.gov/pubmed/?term=Boynton AL%5BAuthor%5D&cauthor=true&cauthor_uid=9397157). Rapid disruption of gap junctional communication and phosphorylation of connexin43 by platelet-derived growth factor in T51B rat liver epithelial cells expressing platelet-derived growth factor receptor. [*J. Cell. Physiol.*](https://www.ncbi.nlm.nih.gov/pubmed/9397157)**174,** 66-77 (1998).
49. [Moorby, C. D](https://www.ncbi.nlm.nih.gov/pubmed/?term=Moorby CD%5BAuthor%5D&cauthor=true&cauthor_uid=10366436). & [Gherardi, E](https://www.ncbi.nlm.nih.gov/pubmed/?term=Gherardi E%5BAuthor%5D&cauthor=true&cauthor_uid=10366436). Expression of a Cx43 deletion mutant in 3T3 A31 fibroblasts prevents PDGF-induced inhibition of cell communication and suppresses cell growth. [*Exp Cell Res.*](https://www.ncbi.nlm.nih.gov/pubmed/?term=C.+D.+Moorby+and+E.+Gherardi%2C+Exp.+Cell+Res.%2C+1999)**249,** 367-376 (1999).
50. [Zhang, X](https://www.ncbi.nlm.nih.gov/pubmed/?term=Zhang X%5BAuthor%5D&cauthor=true&cauthor_uid=29174980). et al. PDGF-mediated PI3K/AKT/β-catenin signaling regulates gap junctions in corpus cavernosum smooth muscle cells. [*Exp Cell Res.*](https://www.ncbi.nlm.nih.gov/pubmed/?term=PDGF-mediated+PI3K%2FAKT%2Fβ-catenin+signaling+regulates+gap+junctions+in+corpus+cavernosum+smooth+muscle+cells) **362,** 252-259 (2018).
51. [Doble, B. W](https://www.ncbi.nlm.nih.gov/pubmed/?term=Doble BW%5BAuthor%5D&cauthor=true&cauthor_uid=7776963). & [Kardami, E](https://www.ncbi.nlm.nih.gov/pubmed/?term=Kardami E%5BAuthor%5D&cauthor=true&cauthor_uid=7776963). Basic fibroblast growth factor stimulates connexin-43 expression and intercellular communication of cardiac fibroblasts. [*Mol. Cell. Biochem*.](https://www.ncbi.nlm.nih.gov/pubmed/7776963) **143**, 81-87 (1995).
52. [Reuss, B](https://www.ncbi.nlm.nih.gov/pubmed/?term=Reuss B%5BAuthor%5D&cauthor=true&cauthor_uid=10756073)., [Hertel, M](https://www.ncbi.nlm.nih.gov/pubmed/?term=Hertel M%5BAuthor%5D&cauthor=true&cauthor_uid=10756073)., [Werner, S](https://www.ncbi.nlm.nih.gov/pubmed/?term=Werner S%5BAuthor%5D&cauthor=true&cauthor_uid=10756073). & [Unsicker, K](https://www.ncbi.nlm.nih.gov/pubmed/?term=Unsicker K%5BAuthor%5D&cauthor=true&cauthor_uid=10756073). Fibroblast growth factors-5 and -9 distinctly regulate expression and function of the gap junction protein connexin43 in cultured astroglial cells from different brain regions. [*Glia*.](https://www.ncbi.nlm.nih.gov/pubmed/?term=Fibroblast+growth+factors-5+and+-9+distinctly+regulate+expression+and+function+of+the+gap+junction+protein+connexin43+in+cultured+astroglial+cells+from+different+brain+regions.) **30**, 231-241(2000).
53. [Schalper, K. A](https://www.ncbi.nlm.nih.gov/pubmed/?term=Schalper KA%5BAuthor%5D&cauthor=true&cauthor_uid=22218428). et al. Modulation of gap junction channels and hemichannels by growth factors. [*Mol. Biosyst*.](https://www.ncbi.nlm.nih.gov/pubmed/22218428) **8**, 685-698 (2012).
54. [Sakurai, T](https://www.ncbi.nlm.nih.gov/pubmed/?term=Sakurai T%5BAuthor%5D&cauthor=true&cauthor_uid=23742896)., [Tsuchida, M](https://www.ncbi.nlm.nih.gov/pubmed/?term=Tsuchida M%5BAuthor%5D&cauthor=true&cauthor_uid=23742896)., [Lampe, P. D](https://www.ncbi.nlm.nih.gov/pubmed/?term=Lampe PD%5BAuthor%5D&cauthor=true&cauthor_uid=23742896). & [Murakami, M](https://www.ncbi.nlm.nih.gov/pubmed/?term=Murakami M%5BAuthor%5D&cauthor=true&cauthor_uid=23742896). Cardiomyocyte FGF signaling is required for Cx43 phosphorylation and cardiac gap junction maintenance. [*Exp Cell Res.*](https://www.ncbi.nlm.nih.gov/pubmed/23742896) **319**, 2152-2165 (2013).
55. [Reuss, B](https://www.ncbi.nlm.nih.gov/pubmed/?term=Reuss B%5BAuthor%5D&cauthor=true&cauthor_uid=10756073)., [Hertel, M](https://www.ncbi.nlm.nih.gov/pubmed/?term=Hertel M%5BAuthor%5D&cauthor=true&cauthor_uid=10756073)., [Werner, S](https://www.ncbi.nlm.nih.gov/pubmed/?term=Werner S%5BAuthor%5D&cauthor=true&cauthor_uid=10756073). & [Unsicker, K](https://www.ncbi.nlm.nih.gov/pubmed/?term=Unsicker K%5BAuthor%5D&cauthor=true&cauthor_uid=10756073). Fibroblast growth factors-5 and -9 distinctly regulate expression and function of the gap junction protein connexin43 in cultured astroglial cells from different brain regions. [*Glia.*](https://www.ncbi.nlm.nih.gov/pubmed/?term=B.+Reuss%2C+M.+Hertel%2C+S.+Werner+and+K.+Unsicker%2C+Glia) **30,** 231-241 (2000).
56. [Liu, X. Y](https://www.ncbi.nlm.nih.gov/pubmed/?term=Liu XY%5BAuthor%5D&cauthor=true&cauthor_uid=30287900). et al. FGF-7 Dictates Osteocyte Cell Processes Through Beta-Catenin Transduction. [*Sci Rep*.](https://www.ncbi.nlm.nih.gov/pubmed/?term=FGF-7+Dictates+Osteocyte+Cell+Processes+Through+Beta-Catenin+Transduction.) **8**, 14792 (2018).
57. [Liu, W](https://www.ncbi.nlm.nih.gov/pubmed/?term=Liu W%5BAuthor%5D&cauthor=true&cauthor_uid=31666990). et al. TGF-β1 facilitates cell-cell communication in osteocytes via connexin43- and pannexin1-dependent gap junctions. [*Cell Death Discov*.](https://www.ncbi.nlm.nih.gov/pubmed/?term=TGF-beta1+facilitates+cell-cell+communication+in+osteocytes+via+connexin43-+and+pannexin1-dependent+gap+junctions.) [**5**, 141](https://doi.org/10.1038/s41420-019-0221-3) (2019).
58. [Wang, Q](https://www.ncbi.nlm.nih.gov/pubmed/?term=Wang Q%5BAuthor%5D&cauthor=true&cauthor_uid=30897973). et al. The involvement of the ERK-MAPK pathway in TGF-β1-mediated connexin43-gap junction formation in chondrocytes. [*Connect. Tissue Res.*](https://www.ncbi.nlm.nih.gov/pubmed/?term=The+involvement+of+the+ERK-MAPK+pathway+in+TGF-β1–mediated+connexin43-gap+junction+formation+in+chondrocytes) **60**, 477-486 (2019).
59. [Wang, Q](https://www.ncbi.nlm.nih.gov/pubmed/?term=Wang Q%5BAuthor%5D&cauthor=true&cauthor_uid=30444057). et al. TGF-β1 promotes gap junctions formation in chondrocytes via Smad3/Smad4 signalling. *Cell Prolif.* **52**, e12544 (2018).
60. Chen YC, Chang HM, Cheng JC, Tsai HD, Wu CH, Leung PC. Transforming growth factor-beta1 up-regulates connexin43 expression in human granulosa cells. *Hum. Reprod.* **30**, 2190-2201 (2015).
61. Cheng JC, Chang HM, Fang L, Sun YP, Leung PC. TGF-beta1 up-regulates connexin43 expression: a potential mechanism for human trophoblast cell differentiation. *J. Cell. Physiol.* **230**,1558-1566 (2015).
62. Lim MC, Maubach G, Zhuo L. TGF-beta1 down-regulates connexin 43 expression and gap junction intercellular communication in rat hepatic stellate cells. *Eur. J. Cell Biol.* **88**,719-730 (2009).
63. Neuhaus J, Heinrich M, Schwalenberg T, Stolzenburg J-U. TGF-β1 Inhibits Cx43 Expression and Formation of Functional Syncytia in Cultured Smooth Muscle Cells from Human Detrusor. *Eur. Urol.* **55**,491-498 (2009).
64. [Theodossiou, S. K](https://www.ncbi.nlm.nih.gov/pubmed/?term=Theodossiou SK%5BAuthor%5D&cauthor=true&cauthor_uid=30538046)., [Tokle, J](https://www.ncbi.nlm.nih.gov/pubmed/?term=Tokle J%5BAuthor%5D&cauthor=true&cauthor_uid=30538046). & [Schiele, N. R](https://www.ncbi.nlm.nih.gov/pubmed/?term=Schiele NR%5BAuthor%5D&cauthor=true&cauthor_uid=30538046). TGFβ2-induced tenogenesis impacts cadherin and connexin cell-cell junction proteins in mesenchymal stem cells. *Biochem. Biophys. Res. Commun.* **508**, 889-893 (2019).
65. Kabir, N., Chaturvedi, K., Liu, L. S. & Sarkar, D. K. [Transforming growth factor-beta3 increases gap-junctional communication among folliculostellate cells to release basic fibroblast growth factor.](https://www.ncbi.nlm.nih.gov/pubmed/15961560) *Endocrinology*. **146,** 4054-4060 (2005).
66. [Hervé, J. C](https://www.ncbi.nlm.nih.gov/pubmed/?term=Hervé JC%5BAuthor%5D&cauthor=true&cauthor_uid=22197781), [Derangeon, M](https://www.ncbi.nlm.nih.gov/pubmed/?term=Derangeon M%5BAuthor%5D&cauthor=true&cauthor_uid=22197781)., [Sarrouilhe, D](https://www.ncbi.nlm.nih.gov/pubmed/?term=Sarrouilhe D%5BAuthor%5D&cauthor=true&cauthor_uid=22197781)., [Giepmans, B. N](https://www.ncbi.nlm.nih.gov/pubmed/?term=Giepmans BN%5BAuthor%5D&cauthor=true&cauthor_uid=22197781). & [Bourmeyster, N](https://www.ncbi.nlm.nih.gov/pubmed/?term=Bourmeyster N%5BAuthor%5D&cauthor=true&cauthor_uid=22197781). Gap junctional channels are parts of multiprotein complexes. [*Biochim. Biophys. Acta*.](https://www.ncbi.nlm.nih.gov/pubmed/?term=Gap+junctional+channels+are+parts+of+multiprotein+complexes.) **1818**, 1844-1865 (2012).
67. [Squecco, R](https://www.ncbi.nlm.nih.gov/pubmed/?term=Squecco R%5BAuthor%5D&cauthor=true&cauthor_uid=16957055). et al. Sphingosine 1-phosphate induces myoblast differentiation through Cx43 protein expression: a role for a gap junction-dependent and -independent function. [*Mol. Biol. Cell*.](https://www.ncbi.nlm.nih.gov/pubmed/?term=Sphingosine+1-phosphate+induces+myoblast+differentiation+through+Cx43+protein+expression%3A+a+role+for+a+gap+junction-dependent+and+-independent+function.) **17**, 4896-4910 (2006).
68. [Giepmans, B. N](https://www.ncbi.nlm.nih.gov/pubmed/?term=Giepmans BN%5BAuthor%5D&cauthor=true&cauthor_uid=12064592)., [Verlaan, I](https://www.ncbi.nlm.nih.gov/pubmed/?term=Verlaan I%5BAuthor%5D&cauthor=true&cauthor_uid=12064592). & [Moolenaar, W. H](https://www.ncbi.nlm.nih.gov/pubmed/?term=Moolenaar WH%5BAuthor%5D&cauthor=true&cauthor_uid=12064592). Connexin-43 interactions with ZO-1 and alpha- and beta-tubulin. [*Cell Commun. Adhes*.](https://www.ncbi.nlm.nih.gov/pubmed/?term=Connexin-43+interactions+with+ZO-1+and+alpha-+and+beta-tubulin.) **8**, 219-223 (2001).
69. Paris, L., Tonutti, L., Vannini, C. & Bazzoni, G. Structural organization of the tight junctions. *Biochim. Biophys. Acta*. **1778**, 646-659 (2008).
70. [Singh, D](https://www.ncbi.nlm.nih.gov/pubmed/?term=Singh D%5BAuthor%5D&cauthor=true&cauthor_uid=15980428)., [Solan, J. L](https://www.ncbi.nlm.nih.gov/pubmed/?term=Solan JL%5BAuthor%5D&cauthor=true&cauthor_uid=15980428)., [Taffet, S. M](https://www.ncbi.nlm.nih.gov/pubmed/?term=Taffet SM%5BAuthor%5D&cauthor=true&cauthor_uid=15980428)., [Javier, R](https://www.ncbi.nlm.nih.gov/pubmed/?term=Javier R%5BAuthor%5D&cauthor=true&cauthor_uid=15980428). & [Lampe, P. D](https://www.ncbi.nlm.nih.gov/pubmed/?term=Lampe PD%5BAuthor%5D&cauthor=true&cauthor_uid=15980428). Connexin 43 interacts with zona occludens-1 and -2 proteins in a cell cycle stage-specific manner. [*J. Biol. Chem*.](https://www.ncbi.nlm.nih.gov/pubmed/?term=Connexin+43+interacts+with+zona+occludens-1+and+-2+proteins+in+a+cell+cycle+stage-specific+manner.) **280**, 30416-30421 (2005).
71. Toyofuku, T. et al. c-Src regulates the interaction between connexin-43 and ZO-1 in cardiac myocytes. *J. Biol. Chem*. **276**, 1780-1788 (2001).
72. Azarnia, R., Reddy, S., Kmiecik, T. E., Shalloway, D. & Loewenstein, W. R. The cellular src gene product regulates junctional cell-to-cell communication. *Science*. **239**, 398-401(1988).
73. [Kojima, T](https://www.ncbi.nlm.nih.gov/pubmed/?term=Kojima T%5BAuthor%5D&cauthor=true&cauthor_uid=10581193). et al. Induction of tight junctions in human connexin 32 (hCx32)-transfected mouse hepatocytes: connexin 32 interacts with occludin. [*Biochem. Biophys. Res. Commun*.](https://www.ncbi.nlm.nih.gov/pubmed/?term=Induction+of+tight+junctions+in+human+connexin+32+(hCx32)-transfected+mouse+hepatocytes%3A+connexin+32+interacts+with+occludin.) **266**, 222-229 (1999).
74. [Nusrat, A](https://www.ncbi.nlm.nih.gov/pubmed/?term=Nusrat A%5BAuthor%5D&cauthor=true&cauthor_uid=10887180)., [Chen, J. A](https://www.ncbi.nlm.nih.gov/pubmed/?term=Chen JA%5BAuthor%5D&cauthor=true&cauthor_uid=10887180). & [Foley, C. S](https://www.ncbi.nlm.nih.gov/pubmed/?term=Foley CS%5BAuthor%5D&cauthor=true&cauthor_uid=10887180). The coiled-coil domain of occludin can act to organize structural and functional elements of the epithelial tight junction. [*J. Biol. Chem*.](https://www.ncbi.nlm.nih.gov/pubmed/?term=The+coiled-coil+domain+of+occludin+can+act+to+organize+structural+and+functional+elements+of+the+epithelial+tight+junction) **275**, 29816-29822 (2000).
75. [Kojima, T](https://www.ncbi.nlm.nih.gov/pubmed/?term=Kojima T%5BAuthor%5D&cauthor=true&cauthor_uid=11161718). et al. Cx32 but not Cx26 is associated with tight junctions in primary cultures of rat hepatocytes. [*Exp. Cell Res*.](https://www.ncbi.nlm.nih.gov/pubmed/11161718) **263**, 193-201 (2001).
76. [Nagasawa, K](https://www.ncbi.nlm.nih.gov/pubmed/?term=Nagasawa K%5BAuthor%5D&cauthor=true&cauthor_uid=16547974). et al. Possible involvement of gap junctions in the barrier function of tight junctions of brain and lung endothelial cells. [*J. Cell. Physiol.*](https://www.ncbi.nlm.nih.gov/pubmed/?term=Possible+involvement+of+gap+junctions+in+the+barrier+function+of+tight+junctions+of+brain+and+lung+endothelial+cells.) **208**, 123-132 (2006).
77. [Wei, C. J](https://www.ncbi.nlm.nih.gov/pubmed/?term=Wei CJ%5BAuthor%5D&cauthor=true&cauthor_uid=15741167)., [Francis, R](https://www.ncbi.nlm.nih.gov/pubmed/?term=Francis R%5BAuthor%5D&cauthor=true&cauthor_uid=15741167)., [Xu, X](https://www.ncbi.nlm.nih.gov/pubmed/?term=Xu X%5BAuthor%5D&cauthor=true&cauthor_uid=15741167). & [Lo, C. W](https://www.ncbi.nlm.nih.gov/pubmed/?term=Lo CW%5BAuthor%5D&cauthor=true&cauthor_uid=15741167). Connexin43 associated with an N-cadherin-containing multiprotein complex is required for gap junction formation in NIH3T3 cells. [*J. Biol. Chem*.](https://www.ncbi.nlm.nih.gov/pubmed/?term=Connexin43+associated+with+an+N-cadherin-containing+multiprotein+complex+is+required+for+gap+junction+formation+in+NIH3T3+cells.) **280**, 19925-19936 (2005).
78. [Luo, Y](https://www.ncbi.nlm.nih.gov/pubmed/?term=Luo Y%5BAuthor%5D&cauthor=true&cauthor_uid=12640032). & [Radice, G. L](https://www.ncbi.nlm.nih.gov/pubmed/?term=Radice GL%5BAuthor%5D&cauthor=true&cauthor_uid=12640032). Cadherin-mediated adhesion is essential for myofibril continuity across the plasma membrane but not for assembly of the contractile apparatus. [*J. Cell Sci.*](https://www.ncbi.nlm.nih.gov/pubmed/?term=Cadherin-mediated+adhesion+is+essential+for+myofibril+continuity+across+the+plasma+membrane+but+not+for+assembly+of+the+contractile+apparatus.) **116**,14711-14719 (2003).
79. [Govindarajan, R](https://www.ncbi.nlm.nih.gov/pubmed/?term=Govindarajan R%5BAuthor%5D&cauthor=true&cauthor_uid=20881055). et al. Assembly of connexin43 into gap junctions is regulated differentially by E-cadherin and N-cadherin in rat liver epithelial cells. [*Mol. Biol. Cell*.](https://www.ncbi.nlm.nih.gov/pubmed/?term=Assembly+of+connexin43+into+gap+junctions+is+regulated+differentially+by+E-cadherin+and+N-cadherin+in+rat+liver+epithelial+cells.) **21**, 4089-4107 (2010).
80. [Malhotra, J. D](https://www.ncbi.nlm.nih.gov/pubmed/?term=Malhotra JD%5BAuthor%5D&cauthor=true&cauthor_uid=15272007)., [Thyagarajan, V](https://www.ncbi.nlm.nih.gov/pubmed/?term=Thyagarajan V%5BAuthor%5D&cauthor=true&cauthor_uid=15272007)., [Chen, C](https://www.ncbi.nlm.nih.gov/pubmed/?term=Chen C%5BAuthor%5D&cauthor=true&cauthor_uid=15272007). & [Isom, L. L](https://www.ncbi.nlm.nih.gov/pubmed/?term=Isom LL%5BAuthor%5D&cauthor=true&cauthor_uid=15272007). Tyrosine-phosphorylated and nonphosphorylated sodium channel beta1 subunits are differentially localized in cardiac myocytes. [*J. Biol. Chem*.](https://www.ncbi.nlm.nih.gov/pubmed/?term=Tyrosine-phosphorylated+and+nonphosphorylated+sodium+channel+beta1+subunits+are+differentially+localized+in+cardiac+myocytes.) **279**, 40748-40754 (2004).
81. [Liu, J](https://www.ncbi.nlm.nih.gov/pubmed/?term=Liu J%5BAuthor%5D&cauthor=true&cauthor_uid=21172802)., [Xu, J](https://www.ncbi.nlm.nih.gov/pubmed/?term=Xu J%5BAuthor%5D&cauthor=true&cauthor_uid=21172802)., [Gu, S](https://www.ncbi.nlm.nih.gov/pubmed/?term=Gu S%5BAuthor%5D&cauthor=true&cauthor_uid=21172802)., [Nicholson, B. J](https://www.ncbi.nlm.nih.gov/pubmed/?term=Nicholson BJ%5BAuthor%5D&cauthor=true&cauthor_uid=21172802). & [Jiang, J. X](https://www.ncbi.nlm.nih.gov/pubmed/?term=Jiang JX%5BAuthor%5D&cauthor=true&cauthor_uid=21172802). Aquaporin 0 enhances gap junction coupling via its cell adhesion function and interaction with connexin 50. [*J. Cell. Sci.*](https://www.ncbi.nlm.nih.gov/pubmed/?term=Aquaporin+0+enhances+gap+junction+coupling+via+its+cell+adhesion+function+and+interaction+with+connexin+50.) **124**, 198-206 (2011).
82. [Leithe, E](https://www.ncbi.nlm.nih.gov/pubmed/?term=Leithe E%5BAuthor%5D&cauthor=true&cauthor_uid=28526583)., [Mesnil, M](https://www.ncbi.nlm.nih.gov/pubmed/?term=Mesnil M%5BAuthor%5D&cauthor=true&cauthor_uid=28526583). & [Aasen, T](https://www.ncbi.nlm.nih.gov/pubmed/?term=Aasen T%5BAuthor%5D&cauthor=true&cauthor_uid=28526583). The connexin 43 C-terminus: A tail of many tales. [*Biochim. Biophys. Acta. Biomembr*.](https://www.ncbi.nlm.nih.gov/pubmed/?term=The+connexin+43+C-terminus%3A+A+tail+of+many+tales.) **1860**, 48-64 (2018).
83. Park, D. J. et al. Akt phosphorylates Connexin43 on Ser373, a "mode-1" binding site for 14-3-3. *Cell Commun. Adhes*. **14**, 211-226 (2007).
84. [Johnstone, S. R](https://www.ncbi.nlm.nih.gov/pubmed/?term=Johnstone SR%5BAuthor%5D&cauthor=true&cauthor_uid=22652908). et al. MAPK phosphorylation of connexin 43 promotes binding of cyclin E and smooth muscle cell proliferation. [*Circ. Res*.](https://www.ncbi.nlm.nih.gov/pubmed/?term=MAPK+phosphorylation+of+connexin+43+promotes+binding+of+cyclin+E+and+smooth+muscle+cell+proliferation.) **111**, 201-211 (2012).
85. [Lampe, P. D](https://www.ncbi.nlm.nih.gov/pubmed/?term=Lampe PD%5BAuthor%5D&cauthor=true&cauthor_uid=10871288). et al. Phosphorylation of connexin43 on serine368 by protein kinase C regulates gap junctional communication. [*J. Cell. Biol.*](https://www.ncbi.nlm.nih.gov/pubmed/10871288)**149**, 1503-1512 (2000).
86. About, I., Proust, J. P., Raffo, S., Mitsiadis, T. A. & Franquin, J. C. In vivo and in vitro expression of connexin 43 in human teeth. *Connect. Tissue Res*. **43**, 232-237 (2002).
87. Kagayama, M., Akita, H. & Sasano, Y. Immunohistochemical localization of connexin 43 in the developing tooth germ of rat. *Anat. Embryol (Berl)*. **191**, 561-568 (1995).
88. [Pinero, G. J](https://www.ncbi.nlm.nih.gov/pubmed/?term=Pinero GJ%5BAuthor%5D&cauthor=true&cauthor_uid=7883586)., [Parker, S](https://www.ncbi.nlm.nih.gov/pubmed/?term=Parker S%5BAuthor%5D&cauthor=true&cauthor_uid=7883586)., [Rundus, V](https://www.ncbi.nlm.nih.gov/pubmed/?term=Rundus V%5BAuthor%5D&cauthor=true&cauthor_uid=7883586)., [Hertzberg, E. L](https://www.ncbi.nlm.nih.gov/pubmed/?term=Hertzberg EL%5BAuthor%5D&cauthor=true&cauthor_uid=7883586). & [Minkoff, R](https://www.ncbi.nlm.nih.gov/pubmed/?term=Minkoff R%5BAuthor%5D&cauthor=true&cauthor_uid=7883586). Immunolocalization of connexin 43 in the tooth germ of the neonatal rat. [*Histochem J.*](https://www.ncbi.nlm.nih.gov/pubmed/?term=Immunolocalization+of+connexin+43+in+the+tooth+germ+of+the+neonatal+rat.) **26**, 765-770 (1994).
89. [Murakami, S](https://www.ncbi.nlm.nih.gov/pubmed/?term=Murakami S%5BAuthor%5D&cauthor=true&cauthor_uid=11411311)., [Muramatsu, T](https://www.ncbi.nlm.nih.gov/pubmed/?term=Muramatsu T%5BAuthor%5D&cauthor=true&cauthor_uid=11411311). & [Shimono, M](https://www.ncbi.nlm.nih.gov/pubmed/?term=Shimono M%5BAuthor%5D&cauthor=true&cauthor_uid=11411311). Expression and localization of connexin 43 in rat incisor odontoblasts. [*Anat Embryol (Berl).*](https://www.ncbi.nlm.nih.gov/pubmed/11411311) **203**, 367-374 (2001).
90. [Inai, T](https://www.ncbi.nlm.nih.gov/pubmed/?term=Inai T%5BAuthor%5D&cauthor=true&cauthor_uid=9376177)., [Nakamura, K](https://www.ncbi.nlm.nih.gov/pubmed/?term=Nakamura K%5BAuthor%5D&cauthor=true&cauthor_uid=9376177)., [Kurisu, K](https://www.ncbi.nlm.nih.gov/pubmed/?term=Kurisu K%5BAuthor%5D&cauthor=true&cauthor_uid=9376177). & [Shibata Y](https://www.ncbi.nlm.nih.gov/pubmed/?term=Shibata Y%5BAuthor%5D&cauthor=true&cauthor_uid=9376177). Immunohistochemical localization of connexin43 in the enamel organ of the rat upper incisor during ameloblast development. [*Arch. Histol. Cytol.*](https://www.ncbi.nlm.nih.gov/pubmed/?term=Immunohistochemical+localization+of+connexin43+in+the+enamel+organ+of+the+rat+upper+incisor+during+ameloblast+development) **60**, 297-306 (1997).
91. Al-Ansari, S. et al. The Importance of Connexin 43 in Enamel Development and Mineralization. *Front. Physiol.* <https://doi.org/10.3389/fphys.2018.00750> (2018).
92. [Toth, K](https://www.ncbi.nlm.nih.gov/pubmed/?term=Toth K%5BAuthor%5D&cauthor=true&cauthor_uid=20127707)., [Shao, Q](https://www.ncbi.nlm.nih.gov/pubmed/?term=Shao Q%5BAuthor%5D&cauthor=true&cauthor_uid=20127707)., [Lorentz, R](https://www.ncbi.nlm.nih.gov/pubmed/?term=Lorentz R%5BAuthor%5D&cauthor=true&cauthor_uid=20127707). & [Laird, D.W](https://www.ncbi.nlm.nih.gov/pubmed/?term=Laird DW%5BAuthor%5D&cauthor=true&cauthor_uid=20127707). Decreased levels of Cx43 gap junctions result in ameloblast dysregulation and enamel hypoplasia in Gja1Jrt/+ mice. [*J. Cell. Physiol.*](https://www.ncbi.nlm.nih.gov/pubmed/?term=Decreased+levels+of+Cx43+gap+junctions+result+in+ameloblast+dysregulation+and+enamel+hypoplasia+in+Gja1Jrt%2F%2B+mice.) **223**, 601-609 (2010).
93. [Uribe, P](https://www.ncbi.nlm.nih.gov/pubmed/?term=Uribe P%5BAuthor%5D&cauthor=true&cauthor_uid=30216610). et al. Study on site-specific expression of bone formation and resorption factors in human dental follicles. [*Eur. J. Oral Sci.*](https://www.ncbi.nlm.nih.gov/pubmed/?term=Study+on+site-specific+expression+of+bone+formation+and+resorption+factors+in+human+dental+follicles) **126**, 439-448 (2018).
94. [Fried, K](https://www.ncbi.nlm.nih.gov/pubmed/?term=Fried K%5BAuthor%5D&cauthor=true&cauthor_uid=8946246)., [Mitsiadis, T. A](https://www.ncbi.nlm.nih.gov/pubmed/?term=Mitsiadis TA%5BAuthor%5D&cauthor=true&cauthor_uid=8946246)., [Guerrier, A](https://www.ncbi.nlm.nih.gov/pubmed/?term=Guerrier A%5BAuthor%5D&cauthor=true&cauthor_uid=8946246)., [Haegerstrand, A](https://www.ncbi.nlm.nih.gov/pubmed/?term=Haegerstrand A%5BAuthor%5D&cauthor=true&cauthor_uid=8946246). & [Meister, B](https://www.ncbi.nlm.nih.gov/pubmed/?term=Meister B%5BAuthor%5D&cauthor=true&cauthor_uid=8946246). Combinatorial expression patterns of the connexins 26, 32, and 43 during development, homeostasis, and regeneration of rat teeth. [*Int. J. Dev. Biol*.](https://www.ncbi.nlm.nih.gov/pubmed/?term=Combinatorial+expression+patterns+of+the+connexins+26%2C+32) **40**, 985-995 (1996).
95. [Amano, K](https://www.ncbi.nlm.nih.gov/pubmed/?term=Amano K%5BAuthor%5D&cauthor=true&cauthor_uid=22699666). et al. Cleft lip in oculodentodigital dysplasia suggests novel roles for connexin43. [*J. Dent. Res.*](https://www.ncbi.nlm.nih.gov/pubmed/?term=Cleft+lip+in+oculodentodigital+dysplasia+suggests+novel+roles+for+connexin43) **91**, 38S-44S (2012).
96. [Yamada, A](https://www.ncbi.nlm.nih.gov/pubmed/?term=Yamada A%5BAuthor%5D&cauthor=true&cauthor_uid=26565022). et al. Connexin 43 Is Necessary for Salivary Gland Branching Morphogenesis and FGF10-induced ERK1/2 Phosphorylation. [*J. Biol. Chem*.](https://www.ncbi.nlm.nih.gov/pubmed/?term=Connexin+43+Is+Necessary+for+Salivary+Gland+Branching+Morphogenesis+and+FGF10-induced+ERK1%2F2+Phosphorylation.) **291**, 904-912 (2016).
97. Ihara, A., Muramatsu, T. & Shimono, M. Expression of connexin 32 and 43 in developing rat submandibular salivary glands. [*Arch. Oral Biol*.](https://www.ncbi.nlm.nih.gov/pubmed/10761876) **45**, 227-235 (2000).
98. [Kim, J. Y](https://www.ncbi.nlm.nih.gov/pubmed/?term=Kim JY%5BAuthor%5D&cauthor=true&cauthor_uid=15846511)., [Cho, S. W](https://www.ncbi.nlm.nih.gov/pubmed/?term=Cho SW%5BAuthor%5D&cauthor=true&cauthor_uid=15846511). & [Lee, M. J](https://www.ncbi.nlm.nih.gov/pubmed/?term=Lee MJ%5BAuthor%5D&cauthor=true&cauthor_uid=15846511). Inhibition of connexin 43 alters Shh and Bmp-2 expression patterns in embryonic mouse tongue. [*Cell Tissue Res*.](https://www.ncbi.nlm.nih.gov/pubmed/?term=Inhibition+of+connexin+43+alters+Shh+and+Bmp-2+expression+patterns+in+embryonic+mouse+tongue.) **320**, 409-415 (2005).
99. [Lee, M. J](https://www.ncbi.nlm.nih.gov/pubmed/?term=Lee MJ%5BAuthor%5D&cauthor=true&cauthor_uid=16552524). et al. Association of Shh and Ptc with keratin localization in the initiation of the formation of circumvallate papilla and von Ebner's gland. [*Cell Tissue Res.*](https://www.ncbi.nlm.nih.gov/pubmed/?term=Association+of+Shh+and+Ptc+with+keratin+localization+in+the+initiation+of+the+formation+of+circumvallate+papilla+and+von+Ebner's+gland) **325**, 253-261 (2006).

100. [Kato, R](https://www.ncbi.nlm.nih.gov/pubmed/?term=Kato R%5BAuthor%5D&cauthor=true&cauthor_uid=23677649). et al. Gap-junction-mediated communication in human periodontal ligament cells. [*J. Dent. Res*.](https://www.ncbi.nlm.nih.gov/pubmed/?term=Gap-junction-mediated+communication+in+human+periodontal+ligament+cells) **92**, 635-640 (2013).

101. [Yamaoka, Y](https://www.ncbi.nlm.nih.gov/pubmed/?term=Yamaoka Y%5BAuthor%5D&cauthor=true&cauthor_uid=11145016). et al. Double expressions of connexin 43 and 32 in human periodontal ligament fibroblasts. [*Tissue Cell.*](https://www.ncbi.nlm.nih.gov/pubmed/?term=Double+expressions+of+connexin+43+and+32+in+human+periodontal+ligament+fibroblasts.) **32**, 328-335 (2000).

102. [Li, S](https://www.ncbi.nlm.nih.gov/pubmed/?term=Li S%5BAuthor%5D&cauthor=true&cauthor_uid=25731708). et al. Connexin 43 and ERK regulate tension-induced signal transduction in human periodontal ligament fibroblasts. [*J. Orthop. Res.*](https://www.ncbi.nlm.nih.gov/pubmed/?term=Connexin+43+and+ERK+regulate+tension-induced+signal+transduction+in+human+periodontal+ligament+fibroblasts) **33**, 1008-1014 (2015).

103. [Yamaoka, Y](https://www.ncbi.nlm.nih.gov/pubmed/?term=Yamaoka Y%5BAuthor%5D&cauthor=true&cauthor_uid=12441089)., [Sawa, Y](https://www.ncbi.nlm.nih.gov/pubmed/?term=Sawa Y%5BAuthor%5D&cauthor=true&cauthor_uid=12441089)., [Ebata, N](https://www.ncbi.nlm.nih.gov/pubmed/?term=Ebata N%5BAuthor%5D&cauthor=true&cauthor_uid=12441089)., [Ibuki, N](https://www.ncbi.nlm.nih.gov/pubmed/?term=Ibuki N%5BAuthor%5D&cauthor=true&cauthor_uid=12441089). & [Yoshida, S](https://www.ncbi.nlm.nih.gov/pubmed/?term=Yoshida S%5BAuthor%5D&cauthor=true&cauthor_uid=12441089). Cultured periodontal ligament fibroblasts express diverse connexins. [*Tissue Cell*.](https://www.ncbi.nlm.nih.gov/pubmed/?term=Cultured+periodontal+ligament+fibroblasts+express+diverse+connexins.) **34**, 375-380 (2002).

104. [Su, M](https://www.ncbi.nlm.nih.gov/pubmed/?term=Su M%5BAuthor%5D&cauthor=true&cauthor_uid=9207768). et al. Expression of Connexin 43 in Rat Mandibular Bone and Periodontal Ligament (PDL) Cells during Experimental Tooth Movement. [*J. Dent. Res*.](https://www.ncbi.nlm.nih.gov/pubmed/?term=Expression+of+Connexin+43+in+Rat+Mandibular+Bone+and+Periodontal+Ligament+(PDL)+Cells+during+Experimental+Tooth+Movement) **76**, 1357-1366 (1997).

105. [Xu, C](https://www.ncbi.nlm.nih.gov/pubmed/?term=Xu C%5BAuthor%5D&cauthor=true&cauthor_uid=22871357). et al. Cyclic stretch influenced expression of membrane connexin 43 in human periodontal ligament cell. [*Arch. Oral Biol.*](https://www.ncbi.nlm.nih.gov/pubmed/?term=Cyclic+stretch+influenced+expression+of+membrane+connexin+43+in+human+periodontal+ligament+cell.) **57**, 1602-1608 (2012).

106. [Ye, P](https://www.ncbi.nlm.nih.gov/pubmed/?term=Ye P%5BAuthor%5D&cauthor=true&cauthor_uid=10951401)., [Chapple, C. C](https://www.ncbi.nlm.nih.gov/pubmed/?term=Chapple CC%5BAuthor%5D&cauthor=true&cauthor_uid=10951401)., [Kumar, R. K](https://www.ncbi.nlm.nih.gov/pubmed/?term=Kumar RK%5BAuthor%5D&cauthor=true&cauthor_uid=10951401). & [Hunter, N](https://www.ncbi.nlm.nih.gov/pubmed/?term=Hunter N%5BAuthor%5D&cauthor=true&cauthor_uid=10951401). Expression patterns of E-cadherin, involucrin, and connexin gap junction proteins in the lining epithelia of inflamed gingiva. [*J. Pathol*.](https://www.ncbi.nlm.nih.gov/pubmed/?term=Expression+patterns+of+E-cadherin%2C+involucrin%2C+and+connexin+gap+junction+proteins+in+the+lining+epithelia+of) **192**, 58-66 (2000).

107. Hatakeyama, S. et al. Expression pattern of adhesion molecules in junctional epithelium differs from that in other gingival epithelia. J. Periodontal. Res. **41**, 322-328 (2006).

108. [Fujita, T](https://www.ncbi.nlm.nih.gov/pubmed/?term=Fujita T%5BAuthor%5D&cauthor=true&cauthor_uid=18230110). et al. Irsogladine maleate counters the interleukin-1 beta-induced suppression in gap-junctional intercellular communication but does not affect the interleukin-1 beta-induced zonula occludens protein-1 levels in human gingival epithelial cells. [*J. Periodontal. Res.*](https://www.ncbi.nlm.nih.gov/pubmed/?term=Irsogladine+maleate+counters+the+interleukin-1+beta-induced+suppression+in+gap-junctional+intercellular+communication+but+does+not+affect+the+interleukin-1+beta-induced+zonula+occludens+protein-1+levels+in+human+gingival+epithelial+cells.) **43**, 96-102 (2008).

109. [Muramatsu, T](https://www.ncbi.nlm.nih.gov/pubmed/?term=Muramatsu T%5BAuthor%5D&cauthor=true&cauthor_uid=19194037)., [Uekusa, T](https://www.ncbi.nlm.nih.gov/pubmed/?term=Uekusa T%5BAuthor%5D&cauthor=true&cauthor_uid=19194037). & [Masaoka, T](https://www.ncbi.nlm.nih.gov/pubmed/?term=Masaoka T%5BAuthor%5D&cauthor=true&cauthor_uid=19194037). Differential expression and localization of connexins 26 and 43 in the rat gingival epithelium. [*Arch. Histol. Cytol*.](https://www.ncbi.nlm.nih.gov/pubmed/?term=Differential+expression+and+localization+of+connexins+26+and+43+in+the+rat+gingival+epithelium) **71**, 147-154 (2008).

110. [Tarzemany, R](https://www.ncbi.nlm.nih.gov/pubmed/?term=Tarzemany R%5BAuthor%5D&cauthor=true&cauthor_uid=25584940)., [Jiang, G](https://www.ncbi.nlm.nih.gov/pubmed/?term=Jiang G%5BAuthor%5D&cauthor=true&cauthor_uid=25584940)., [Larjava, H](https://www.ncbi.nlm.nih.gov/pubmed/?term=Larjava H%5BAuthor%5D&cauthor=true&cauthor_uid=25584940). & [Häkkinen, L](https://www.ncbi.nlm.nih.gov/pubmed/?term=Häkkinen L%5BAuthor%5D&cauthor=true&cauthor_uid=25584940). Expression and function of connexin 43 in human gingival wound healing and fibroblasts. [*PLoS One*.](https://www.ncbi.nlm.nih.gov/pubmed/25584940) **10**, e0115524. (2015).

111. Ibuki, N., Yamaoka, Y., Sawa, Y., Kawasaki, T. & Yoshida, S. Different expressions of connexin 43 and 32 in the fibroblasts of human dental pulp. [*Tissue Cell.*](https://www.ncbi.nlm.nih.gov/pubmed/?term=Different+expressions+of+connexin+43+and+32+in+the+fibroblasts+of+human+dental+pulp.) **34**, 170-176 (2002).

112. [Chung, C. K](https://www.ncbi.nlm.nih.gov/pubmed/?term=Chung CK%5BAuthor%5D&cauthor=true&cauthor_uid=17450382)., [Muramatsu,T](https://www.ncbi.nlm.nih.gov/pubmed/?term=Muramatsu T%5BAuthor%5D&cauthor=true&cauthor_uid=17450382)., [Uekusa,T](https://www.ncbi.nlm.nih.gov/pubmed/?term=Uekusa T%5BAuthor%5D&cauthor=true&cauthor_uid=17450382)., [Sasaki,H](https://www.ncbi.nlm.nih.gov/pubmed/?term=Sasaki H%5BAuthor%5D&cauthor=true&cauthor_uid=17450382). & [Shimono, M](https://www.ncbi.nlm.nih.gov/pubmed/?term=Shimono M%5BAuthor%5D&cauthor=true&cauthor_uid=17450382). Inhibition of connexin 43 expression and function in cultured rat dental pulp cells by antisense oligonucleotide. [*Cell Tissue Res*.](https://www.ncbi.nlm.nih.gov/pubmed/?term=Inhibition+of+connexin+43+expression+and+function+in+cultured+rat+dental+pulp+cells+by+antisense+oligonucleotide) **329**, 295-300 (2007).

113. [Li, S](https://www.ncbi.nlm.nih.gov/pubmed/?term=Li S%5BAuthor%5D&cauthor=true&cauthor_uid=26376117). et al. Connexin43-containing gap junctions potentiate extracellular Ca(2)(+)-induced odontoblastic differentiation of human dental pulp stem cells via Erk1/2. [*Exp. Cell Res*.](https://www.ncbi.nlm.nih.gov/pubmed/26376117) **338**, 1-9 (2015).

114. [Muramatsu, T](https://www.ncbi.nlm.nih.gov/pubmed/?term=Muramatsu T%5BAuthor%5D&cauthor=true&cauthor_uid=15548271). et al. Reduction of connexin 43 expression in aged human dental pulp. [*Int. Endod. J.*](https://www.ncbi.nlm.nih.gov/pubmed/?term=Reduction+of+connexin+43+expression+in+aged+human+dental+pulp) **37**, 814-818 (2004).

115. [Amano, T](https://www.ncbi.nlm.nih.gov/pubmed/?term=Amano T%5BAuthor%5D&cauthor=true&cauthor_uid=16632756)., [Muramatsu, T](https://www.ncbi.nlm.nih.gov/pubmed/?term=Muramatsu T%5BAuthor%5D&cauthor=true&cauthor_uid=16632756)., [Amemiya, K](https://www.ncbi.nlm.nih.gov/pubmed/?term=Amemiya K%5BAuthor%5D&cauthor=true&cauthor_uid=16632756)., [Kubo, K](https://www.ncbi.nlm.nih.gov/pubmed/?term=Kubo K%5BAuthor%5D&cauthor=true&cauthor_uid=16632756). & [Shimono, M](https://www.ncbi.nlm.nih.gov/pubmed/?term=Shimono M%5BAuthor%5D&cauthor=true&cauthor_uid=16632756). Responses of Rat Pulp Cells to Heat Stress in vitro. [*J. Dent. Res*.](https://www.ncbi.nlm.nih.gov/pubmed/?term=Responses+of+Rat+Pulp+Cells+to+Heat+Stress+in+vitro) **85**, 432-435 (2006).

116. [Köling, A](https://www.ncbi.nlm.nih.gov/pubmed/?term=Köling A%5BAuthor%5D&cauthor=true&cauthor_uid=6585119). & [Rask-Andersen, H](https://www.ncbi.nlm.nih.gov/pubmed/?term=Rask-Andersen H%5BAuthor%5D&cauthor=true&cauthor_uid=6585119). Membrane junctions between odontoblasts and associated cells. A freeze-fracture study of the human odontoblastic cell layer with special reference to its nerve supply. [*Acta Odontol. Scand.*](https://www.ncbi.nlm.nih.gov/pubmed/?term=Membrane+junctions+between+odontoblasts+and+associated+cells.+A+freeze-fracture+study+of+the+human+odontoblastic+cell+layer+with+special+reference+to+its+nerve+supply.) **42**, 13-22 (1984).

117. [Muramatsu, T](https://www.ncbi.nlm.nih.gov/pubmed/?term=Muramatsu T%5BAuthor%5D&cauthor=true&cauthor_uid=23922183). et al. Immunoelectron microscopic observation of connexin43 in rat odontoblasts. [*Microsc. Res. Tech*.](https://www.ncbi.nlm.nih.gov/pubmed/?term=Immunoelectron+microscopic+observation+of+connexin43+in+rat+odontoblasts) **76**, 988-991 (2013).

118. [Fried, K](https://www.ncbi.nlm.nih.gov/pubmed/?term=Fried K%5BAuthor%5D&cauthor=true&cauthor_uid=8946246)., [Mitsiadis, T. A](https://www.ncbi.nlm.nih.gov/pubmed/?term=Mitsiadis TA%5BAuthor%5D&cauthor=true&cauthor_uid=8946246)., [Guerrier, A](https://www.ncbi.nlm.nih.gov/pubmed/?term=Guerrier A%5BAuthor%5D&cauthor=true&cauthor_uid=8946246)., [Haegerstrand, A](https://www.ncbi.nlm.nih.gov/pubmed/?term=Haegerstrand A%5BAuthor%5D&cauthor=true&cauthor_uid=8946246). & [Meister, B](https://www.ncbi.nlm.nih.gov/pubmed/?term=Meister B%5BAuthor%5D&cauthor=true&cauthor_uid=8946246). Combinatorial expression patterns of the connexins 26, 32, and 43 during development, homeostasis, and regeneration of rat teeth. [*Int. J. Dev. Biol*.](https://www.ncbi.nlm.nih.gov/pubmed/?term=Combinatorial+expression+patterns+of+the+connexins+26%2C+32) **40**, 985-995 (1996).

119. [Farahani, R. M](https://www.ncbi.nlm.nih.gov/pubmed/?term=Farahani RM%5BAuthor%5D&cauthor=true&cauthor_uid=20802180)., [Nguyen, K. A](https://www.ncbi.nlm.nih.gov/pubmed/?term=Nguyen KA%5BAuthor%5D&cauthor=true&cauthor_uid=20802180), [Simonian, M](https://www.ncbi.nlm.nih.gov/pubmed/?term=Simonian M%5BAuthor%5D&cauthor=true&cauthor_uid=20802180). & [Hunter, N](https://www.ncbi.nlm.nih.gov/pubmed/?term=Hunter N%5BAuthor%5D&cauthor=true&cauthor_uid=20802180). Adaptive calcified matrix response of dental pulp to bacterial invasion is associated with establishment of a network of glial fibrillary acidic protein+/glutamine synthetase+ cells. [*Am. J. Pathol*.](https://www.ncbi.nlm.nih.gov/pubmed/?term=Adaptive+calcified+matrix+response+of+dental+pulp+to+bacterial+invasion+is+associated+with+establishment+of+a+network+of+glial+fibrillary+acidic+protein%2B%2Fglutamine+synthetase%2B+cells) **177**,1901-1914 (2010).

120. [Couve, E](https://www.ncbi.nlm.nih.gov/pubmed/?term=Couve E%5BAuthor%5D&cauthor=true&cauthor_uid=24928097). et al. Reactionary Dentinogenesis and Neuroimmune Response in Dental Caries. [*J. Dent. Res*.](https://www.ncbi.nlm.nih.gov/pubmed/?term=Reactionary+Dentinogenesis+and+Neuroimmune+Response+in+Dental+Caries) **93**, 788-793 (2014).

121. [Centeno, V. A](https://www.ncbi.nlm.nih.gov/pubmed/?term=Centeno VA%5BAuthor%5D&cauthor=true&cauthor_uid=25438100), [Fontanetti, P. A](https://www.ncbi.nlm.nih.gov/pubmed/?term=Fontanetti PA%5BAuthor%5D&cauthor=true&cauthor_uid=25438100), [Interlandi, V](https://www.ncbi.nlm.nih.gov/pubmed/?term=Interlandi V%5BAuthor%5D&cauthor=true&cauthor_uid=25438100)., [Ponce, R. H](https://www.ncbi.nlm.nih.gov/pubmed/?term=Ponce RH%5BAuthor%5D&cauthor=true&cauthor_uid=25438100). & [Gallará, R.V](https://www.ncbi.nlm.nih.gov/pubmed/?term=Gallará RV%5BAuthor%5D&cauthor=true&cauthor_uid=25438100). Fluoride alters connexin expression in rat incisor pulp. [*Arch. Oral Biol.*](https://www.ncbi.nlm.nih.gov/pubmed/?term=Fluoride+alters+connexin+expression+in+rat+incisor+pulp) **60**, 313-319 (2015).

122. [Azarnia, R](https://www.ncbi.nlm.nih.gov/pubmed/?term=Azarnia R%5BAuthor%5D&cauthor=true&cauthor_uid=24177450). & [Loewenstein, W. R](https://www.ncbi.nlm.nih.gov/pubmed/?term=Loewenstein WR%5BAuthor%5D&cauthor=true&cauthor_uid=24177450). Intercellular communication and tissue growth : V. A cancer cell strain that fails to make permeable membrane junctions with normal cells. [*J. Membr. Biol.*](https://www.ncbi.nlm.nih.gov/pubmed/?term=Intercellular+Communication+and+Tissue+Growth，1971) **6**, 368-385 (1971).

123. Trosko, J. E., Chang, C. C., Upham, B. L. & Tai, M. H. Ignored hallmarks of carcinogenesis: stem cells and cell-cell communication. [*Ann N Y Acad Sci*.](https://www.ncbi.nlm.nih.gov/pubmed/15650245) **1028**,192-201 (2004).

124. [McLachlan, E](https://www.ncbi.nlm.nih.gov/pubmed/?term=McLachlan E%5BAuthor%5D&cauthor=true&cauthor_uid=17047050)., [Shao, Q](https://www.ncbi.nlm.nih.gov/pubmed/?term=Shao Q%5BAuthor%5D&cauthor=true&cauthor_uid=17047050)., [Wang, H. L](https://www.ncbi.nlm.nih.gov/pubmed/?term=Wang HL%5BAuthor%5D&cauthor=true&cauthor_uid=17047050)., [Langlois, S](https://www.ncbi.nlm.nih.gov/pubmed/?term=Langlois S%5BAuthor%5D&cauthor=true&cauthor_uid=17047050). & [Laird, D.W](https://www.ncbi.nlm.nih.gov/pubmed/?term=Laird DW%5BAuthor%5D&cauthor=true&cauthor_uid=17047050). Connexins act as tumor suppressors in three-dimensional mammary cell organoids by regulating differentiation and angiogenesis. [*Cancer Res.*](https://www.ncbi.nlm.nih.gov/pubmed/?term=Connexins+act+as+tumor+suppressors+in+three-dimensional+mammary+cell+organoids+by+regulating+differentiation+and+angiogenesis.) **66**, 9886-9894 (2006).

125. Sirnes, S. et al. Connexins in colorectal cancer pathogenesis. *Int. J. Cancer.* **137**, 1-11 (2015).

126. Czyż, J., Szpak, K. & Madeja, Z. The role of connexins in prostate cancer promotion and progression. *Nat. Rev. Urol*. **9**, 274-282 (2012).

127. [Yost, S](https://www.ncbi.nlm.nih.gov/pubmed/?term=Yost S%5BAuthor%5D&cauthor=true&cauthor_uid=30420594). et al. Increased virulence of the oral microbiome in oral squamous cell carcinoma revealed by metatranscriptome analyses. [*Int. J. Oral Sci*.](https://www.ncbi.nlm.nih.gov/pubmed/30420594) [**10**,](https://doi.org/10.1038/s41368-018-0037-7) 32 (2018).

128. [Brockmeyer, P](https://www.ncbi.nlm.nih.gov/pubmed/?term=Brockmeyer P%5BAuthor%5D&cauthor=true&cauthor_uid=24788723)., [Jung, K](https://www.ncbi.nlm.nih.gov/pubmed/?term=Jung K%5BAuthor%5D&cauthor=true&cauthor_uid=24788723)., [Perske, C](https://www.ncbi.nlm.nih.gov/pubmed/?term=Perske C%5BAuthor%5D&cauthor=true&cauthor_uid=24788723)., [Schliephake, H](https://www.ncbi.nlm.nih.gov/pubmed/?term=Schliephake H%5BAuthor%5D&cauthor=true&cauthor_uid=24788723). & [Hemmerlein, B](https://www.ncbi.nlm.nih.gov/pubmed/?term=Hemmerlein B%5BAuthor%5D&cauthor=true&cauthor_uid=24788723). Membrane connexin 43 acts as an independent prognostic marker in oral squamous cell carcinoma. [*Int. J. Oncol*.](https://www.ncbi.nlm.nih.gov/pubmed/?term=Membrane+connexin+43+acts+as+an+independent+prognostic+marker+in+oral+squamous+cell+carcinoma.) **45**, 273-281(2014).

129. [Essa, A. A](https://www.ncbi.nlm.nih.gov/pubmed/?term=Essa AA%5BAuthor%5D&cauthor=true&cauthor_uid=27020496)., [Yamazaki, M](https://www.ncbi.nlm.nih.gov/pubmed/?term=Yamazaki M%5BAuthor%5D&cauthor=true&cauthor_uid=27020496). & [Maruyama, S](https://www.ncbi.nlm.nih.gov/pubmed/?term=Maruyama S%5BAuthor%5D&cauthor=true&cauthor_uid=27020496). Tumour-associated macrophages are recruited and differentiated in the neoplastic stroma of oral squamous cell carcinoma. [*Pathology*.](https://www.ncbi.nlm.nih.gov/pubmed/?term=Tumour-associated+macrophages+are+recruited+and+differentiated+in+the+neoplastic+stroma+of+oral+squamous+cell+carcinoma) **48**, 219-227 (2016).

130. [Zhong, W. Q](https://www.ncbi.nlm.nih.gov/pubmed/?term=Zhong WQ%5BAuthor%5D&cauthor=true&cauthor_uid=25270527). et al. Down-regulation of connexin43 and connexin32 in keratocystic odontogenic tumours: potential association with clinical features. [*Histopathology*.](https://www.ncbi.nlm.nih.gov/pubmed/?term=Down-regulation+of+connexin43+and+connexin32+in+keratocystic+odontogenic+tumours%3A+potential+association+with+clinical+features) **66**, 798-807 (2015).

131. [Saitoh, M](https://www.ncbi.nlm.nih.gov/pubmed/?term=Saitoh M%5BAuthor%5D&cauthor=true&cauthor_uid=9230274)., [Oyamada, M](https://www.ncbi.nlm.nih.gov/pubmed/?term=Oyamada M%5BAuthor%5D&cauthor=true&cauthor_uid=9230274)., [Oyamada, Y](https://www.ncbi.nlm.nih.gov/pubmed/?term=Oyamada Y%5BAuthor%5D&cauthor=true&cauthor_uid=9230274)., [Kaku, T](https://www.ncbi.nlm.nih.gov/pubmed/?term=Kaku T%5BAuthor%5D&cauthor=true&cauthor_uid=9230274). & [Mori, M](https://www.ncbi.nlm.nih.gov/pubmed/?term=Mori M%5BAuthor%5D&cauthor=true&cauthor_uid=9230274). Changes in the expression of gap junction proteins (connexins) in hamster tongue epithelium during wound healing and carcinogenesis. [*Carcinogenesis*.](https://www.ncbi.nlm.nih.gov/pubmed/?term=Changes+in+the+expression+of+gap+junction+proteins+(connexins)+in+hamster+tongue+epithelium+during+wound) **18**, 1319-1328 (1997).

132. [Ozawa, H](https://www.ncbi.nlm.nih.gov/pubmed/?term=Ozawa H%5BAuthor%5D&cauthor=true&cauthor_uid=17695503)., [Matsunaga, T](https://www.ncbi.nlm.nih.gov/pubmed/?term=Matsunaga T%5BAuthor%5D&cauthor=true&cauthor_uid=17695503). & [Kamiya, K](https://www.ncbi.nlm.nih.gov/pubmed/?term=Kamiya K%5BAuthor%5D&cauthor=true&cauthor_uid=17695503). Decreased Expression of Connexin-30 and Aberrant Expression of Connexin-26 in Human Head and Neck Cancer. [*Anticancer Res*.](https://www.ncbi.nlm.nih.gov/pubmed/?term=Decreased+Expression+of+Connexin-30+and+Aberrant+Expression+of+Connexin-26+in+Human+Head+and+Neck+Cancer) **27**, 2189-2195 (2007).

133. [Wang, J](https://www.ncbi.nlm.nih.gov/pubmed/?term=Wang J%5BAuthor%5D&cauthor=true&cauthor_uid=23524428)., [Dai, Y](https://www.ncbi.nlm.nih.gov/pubmed/?term=Dai Y%5BAuthor%5D&cauthor=true&cauthor_uid=23524428). & [Huang, Y](https://www.ncbi.nlm.nih.gov/pubmed/?term=Huang Y%5BAuthor%5D&cauthor=true&cauthor_uid=23524428). All-trans retinoic acid restores gap junctional intercellular communication between oral cancer cells with upregulation of Cx32 and Cx43 expressions in vitro. [*Med. Oral Patol. Oral Cir. Bucal.*](https://www.ncbi.nlm.nih.gov/pubmed/?term=All-trans+retinoic+acid+restores+gap+junctional+intercellular+communication+between+oral+cancer+cells+with+upregulation+of+Cx32+and+Cx43+expressions+in+vitro.) **18**, e569-e577 (2013).

134. [Piechocki, M. P](https://www.ncbi.nlm.nih.gov/pubmed/?term=Piechocki MP%5BAuthor%5D&cauthor=true&cauthor_uid=11895921)., [Lonardo, F](https://www.ncbi.nlm.nih.gov/pubmed/?term=Lonardo F%5BAuthor%5D&cauthor=true&cauthor_uid=11895921). & [Ensley, J. F](https://www.ncbi.nlm.nih.gov/pubmed/?term=Ensley JF%5BAuthor%5D&cauthor=true&cauthor_uid=11895921). Anticancer Activity of Docetaxel in Murine Salivary Gland Carcinoma. [*Clin. Cancer Res.*](https://www.ncbi.nlm.nih.gov/pubmed/?term=Anticancer+Activity+of+Docetaxel+in+Murine+Salivary+Gland+Carcinoma) **8**, 870-877 (2002).

135. [Livny, O](https://www.ncbi.nlm.nih.gov/pubmed/?term=Livny O%5BAuthor%5D&cauthor=true&cauthor_uid=12468619). et al. Lycopene Inhibits Proliferation and Enhances Gap-Junction Communication of KB-1 Human Oral Tumor Cell. [*J Nutr.*](https://www.ncbi.nlm.nih.gov/pubmed?term=lycopene inhibits proliferation and enhances gap junctional communication of kb-1 human oral tumor cell&cmd=correctspelling) **132**, 3754-3759 (2002).

136. [Rednam, S](https://www.ncbi.nlm.nih.gov/pubmed/?term=Rednam S%5BAuthor%5D&cauthor=true&cauthor_uid=20829717)., [Hicks, J](https://www.ncbi.nlm.nih.gov/pubmed/?term=Hicks J%5BAuthor%5D&cauthor=true&cauthor_uid=20829717)., [Levy, M. L](https://www.ncbi.nlm.nih.gov/pubmed/?term=Levy ML%5BAuthor%5D&cauthor=true&cauthor_uid=20829717). & [Pappo, A. S](https://www.ncbi.nlm.nih.gov/pubmed/?term=Pappo AS%5BAuthor%5D&cauthor=true&cauthor_uid=20829717). Metastatic Squamous Cell Carcinoma of the Oropharynx in a Child With a Mutation in the Connexin 26 Gene. [*J. Pediatr. Hematol. Oncol*.](https://www.ncbi.nlm.nih.gov/pubmed/?term=Metastatic+Squamous+Cell+Carcinoma+of+the+Oropharynx+in+a+Child+With+a+Mutation+in+the+Connexin+26+Gene.) **33**, 387-389 (2011).

137. [van Steensel, M. A](https://www.ncbi.nlm.nih.gov/pubmed/?term=van Steensel MA%5BAuthor%5D&cauthor=true&cauthor_uid=11918723)., [van, Geel. M](https://www.ncbi.nlm.nih.gov/pubmed/?term=van Geel M%5BAuthor%5D&cauthor=true&cauthor_uid=11918723), [Nahuys, M](https://www.ncbi.nlm.nih.gov/pubmed/?term=Nahuys M%5BAuthor%5D&cauthor=true&cauthor_uid=11918723)., [Smitt, J. H](https://www.ncbi.nlm.nih.gov/pubmed/?term=Smitt JH%5BAuthor%5D&cauthor=true&cauthor_uid=11918723). & [Steijlen, P. M](https://www.ncbi.nlm.nih.gov/pubmed/?term=Steijlen PM%5BAuthor%5D&cauthor=true&cauthor_uid=11918723). A Novel Connexin 26 Mutation in a Patient Diagnosed with Keratitis-Ichthyosis-Deafness Syndrome. [*J. Invest. Dermatol.*](https://www.ncbi.nlm.nih.gov/pubmed/?term=A+Novel+Connexin+26+Mutation+in+a+Patient+Diagnosed+with+Keratitis–Ichthyosis–Deafness+Syndrome.) **118**, 724-727 (2002).

138. [Hadjichristou, C](https://www.ncbi.nlm.nih.gov/pubmed/?term=Hadjichristou C%5BAuthor%5D&cauthor=true&cauthor_uid=28319210). et al. Oculo-Dento-Digital Dysplasia (ODDD) Due to a GJA1 Mutation: Report of a Case with Emphasis on Dental Manifestations. [*Int. J. Prosthodont*.](https://www.ncbi.nlm.nih.gov/pubmed/?term=Oculo-Dento-Digital+Dysplasia+(ODDD)+Due+to+a+GJA1+Mutation%3A+Report+of+a+Case+with+Emphasis+on+Dental+Manifestations.) **30**, 280-285 (2017).

139. [Paznekas, W. A](https://www.ncbi.nlm.nih.gov/pubmed/?term=Paznekas WA%5BAuthor%5D&cauthor=true&cauthor_uid=19338053). et al. GJA1 mutations, variants, and connexin 43 dysfunction as it relates to the oculodentodigital dysplasia phenotype. [*Hum. Mutat.*](https://www.ncbi.nlm.nih.gov/pubmed/?term=GJA1+mutations%2C+variants%2C+and+connexin+43+dysfunction+as+it+relates+to+the+oculodentodigital+dysplasia+phenotype.) **30**, 724-733 (2009).

140. Tarzemany, R., Jiang, G., Jiang, J. X., Larjava, H. & Häkkinen, L. Connexin 43 Hemichannels Regulate the Expression of Wound Healing-Associated Genes in Human Gingival Fibroblasts. *Sci Rep*. **7**, 14157 (2017).

141. [Tarzemany, R](https://www.ncbi.nlm.nih.gov/pubmed/?term=Tarzemany R%5BAuthor%5D&cauthor=true&cauthor_uid=29596891). et al. Connexin 43 regulates the expression of wound healing-related genes in human gingival and skin fibroblasts. [*Exp. Cell Res*.](https://www.ncbi.nlm.nih.gov/pubmed/?term=Connexin+43+regulates+the+expression+of+wound+healing-related+genes+in+human+gingival+and+skin+fibroblasts) **367**, 150-161 (2018).

142. [Li, X](https://www.ncbi.nlm.nih.gov/pubmed/?term=Li X%5BAuthor%5D&cauthor=true&cauthor_uid=31175668). et al. TGF-beta1-Induced Connexin43 Promotes Scar Formation via the Erk/MMP-1/Collagen III Pathway. [*J. Oral Rehabil.*](https://www.ncbi.nlm.nih.gov/pubmed/?term=TGF-beta1-Induced+Connexin43+Promotes+Scar+Formation+via+the+Erk%2FMMP-1%2FCollagen+III+Pathway.) <https://doi.org/10.1111/joor.12829> (2019).

143. [Hsu, J. C](https://www.ncbi.nlm.nih.gov/pubmed/?term=Hsu JC%5BAuthor%5D&cauthor=true&cauthor_uid=21125789). & [Yamada, K. M](https://www.ncbi.nlm.nih.gov/pubmed/?term=Yamada KM%5BAuthor%5D&cauthor=true&cauthor_uid=21125789). Salivary gland branching morphogenesis--recent progress and future opportunities. [*Int. J. Oral Sci*.](https://www.ncbi.nlm.nih.gov/pubmed/21125789) **2**, 117-126 (2010).

144. Shimono, M., Young, Lee. C. & Matsuzaki, H. [Connexins in salivary glands.](https://www.ncbi.nlm.nih.gov/pubmed/10980677) *Eur. J. Morphol*. **38**, 257-261 (2000).

145. [Actis, A. B](https://www.ncbi.nlm.nih.gov/pubmed/?term=Actis AB%5BAuthor%5D&cauthor=true&cauthor_uid=12110338)., [Lampe, P. D](https://www.ncbi.nlm.nih.gov/pubmed/?term=Lampe PD%5BAuthor%5D&cauthor=true&cauthor_uid=12110338). & [Eynard, A. R](https://www.ncbi.nlm.nih.gov/pubmed/?term=Eynard AR%5BAuthor%5D&cauthor=true&cauthor_uid=12110338). Cellular basis and clinical implications of biological markers in salivary tissues their topological distribution in murine submandibular gland. [*Oral Oncol*.](https://www.ncbi.nlm.nih.gov/pubmed/?term=Cellular+basis+and+clinical+implications+of+biological+markers+in+salivary+tissues++their+topological+distribution+in+murine+submandibular+gland) **38**, 441-449 (2002).

146. [Muramatsu, T](https://www.ncbi.nlm.nih.gov/pubmed/?term=Muramatsu T%5BAuthor%5D&cauthor=true&cauthor_uid=8543782)., [Hashimoto, S](https://www.ncbi.nlm.nih.gov/pubmed/?term=Hashimoto S%5BAuthor%5D&cauthor=true&cauthor_uid=8543782). & [Shimono, M](https://www.ncbi.nlm.nih.gov/pubmed/?term=Shimono M%5BAuthor%5D&cauthor=true&cauthor_uid=8543782). Differential Expression of Gap Junction Proteins Connexin32 and 43 in Rat Submandibular and Sublingual Glands . [*J. Histochem. Cytochem.*](https://www.ncbi.nlm.nih.gov/pubmed/?term=Differential+Expression+of+Gap+Junction+Proteins+Connexin32+and+43+in+Rat+Submandibular+and+Sublingual+Glands) **44**, 49-56 (1996).

147. [Kuraoka, A](https://www.ncbi.nlm.nih.gov/pubmed/?term=Kuraoka A%5BAuthor%5D&cauthor=true&cauthor_uid=11894787)., [Yamanaka, I](https://www.ncbi.nlm.nih.gov/pubmed/?term=Yamanaka I%5BAuthor%5D&cauthor=true&cauthor_uid=11894787)., [Miyahara, A](https://www.ncbi.nlm.nih.gov/pubmed/?term=Miyahara A%5BAuthor%5D&cauthor=true&cauthor_uid=11894787)., [Shibata, Y](https://www.ncbi.nlm.nih.gov/pubmed/?term=Shibata Y%5BAuthor%5D&cauthor=true&cauthor_uid=11894787). & [Uemura, T](https://www.ncbi.nlm.nih.gov/pubmed/?term=Uemura T%5BAuthor%5D&cauthor=true&cauthor_uid=11894787). Immunocytochemical studies of major gap junction proteins in rat salivary glands. [*Eur* *Arch Otorhinolaryngol.*](https://www.ncbi.nlm.nih.gov/pubmed/?term=Immunocytochemical+studies+of+major+gap+junction+proteins+in+rat+salivary+glands) **251** Suppl 1: S95-99 (1994).

**Table** 1. The reports on connexins that we can currently collect.

| Protein | Gene name | | Oral cavity-related distribution, development & disorders | References |
| --- | --- | --- | --- | --- |
|  | human | mouse |  |  |
| Cx26 | GJB2 | Gjb2 | gingival  odontoblast  tongue carcinoma  KID  salivary gland | [106][109]  [94]  [131][132]  [136][137]  [144][145] |
| Cx32 | GJB1 | Gjb1 | PDLFs  pulp  keratocystic odontogenic tumors  salivary gland | [103]  [121]  [130]  [144][145][147][97] |
| Cx40 | GJA5 | Gja5 | PDLFs | [103] |
| Cx43 | GJA1 | Gja1 | PDLFs  gingiva  pulp cell; DPSCs; pulp  odontoblast  *ODDD*  tongue  ameloblasts; enamel hypoplasia  HDFCs  OSCC; KCOTs  buccal mucosa  salivary gland | [102][104] [105]  [106][107][108][110][140][141]  [86][112][113][114][115[121]  [116][117][119][120][88]  [95][138][139]  [98][99]  [90][91][92]  [93]  [128][129][133][130]  [142]  [96][147] |
| Cx45 | GJA7 | Gja7 | PDLFs  pulp | [103]  [121] |

Abbreviation: KID: keratitis-ichthyosis-deafness; PDLFs: periodontal ligament fibroblasts; DPSCs: dental pulp stem cells; ODDD: oculodentodigital dysplasia; HDFC: human dental follicle cells; OSCC, oral squamous cell carcinoma; KCOTs: keratocystic odontogenic tumors.

**Figure legends**

**Figure 1. Model of a connexin and gap junction channel**

(A) The connexin monomer. The following abbreviations are used: NT, N-terminus; CL, cytoplasmic loop; CT, C-terminus; EL1 and EL2, extracellular loops 1 and 2; M1-M4, transmembrane domains 1-4. (B) Possible arrangements of connexins in a gap junction channel. The figure shows different components of gap junction channels. Homomeric connexons are formed by a single connexin type. Heteromeric connexons are composed of more than one connexin type. When connexons of the same composition form a gap junction channel, it is classified as a homotypic channel. If the connexons differ in components, the channel is classified as heterotypic.

**Figure 2. Distribution of Cx43 in osteocytes and osteoblasts.**

(A) a. Representative images of IF staining for Cx43 in osteocytes. Immunofluorescence staining was performed with antibodies against connexin 43 (red); nuclei were stained with DAPI (blue). Green represents the FITC-labelled cytoskeleton. Details are shown in the boxed area (white). b. Representative ICC staining of osteocytes. The red arrow indicates the localization of Cx43.

(B) Representative images of IF staining for Cx43 in osteoblasts. a. Representative image of IF staining for Cx43 in primary osteoblasts. b. Representative image of IF staining for Cx43 in the MC3T3 cell line. Immunofluorescence staining was performed with antibodies against connexin 43 (red); nuclei were stained with DAPI (blue). Green represents the FITC-labelled cytoskeleton. Details are shown in the boxed area (white).
